# Nanomicelle‐Based Multi‐mRNA Delivery Promotes Cardiac Repair After Myocardial Infarction

**DOI:** 10.1002/smsc.202500521

**Published:** 2026-05-23

**Authors:** Kazuma Handa, Takuji Kawamura, Yasunobu Mano, Hideyuki Nakanishi, Lisa Fujimura, Chie Kawai, Akima Harada, Kosuke Torigata, Kenji Miki, Keiji Itaka, Shigeru Miyagawa

**Affiliations:** ^1^ Department of Cardiovascular Surgery The University of Osaka Graduate School of Medicine Osaka Japan; ^2^ Clinical Biotechnology Team Center for Infectious Disease Education and Research (CiDER) The University of Osaka Osaka Japan; ^3^ Nucleotide and Peptide Drug Discovery Center Institute of Science Tokyo Tokyo Japan; ^4^ Premium Research Institute for Human Metaverse Medicine (WPI‐PRIMe) The University of Osaka Osaka Japan; ^5^ Institute for Protein Research The University of Osaka Osaka Japan

**Keywords:** heart failure, multigene mRNA therapy, myocardial infarction, pathological remodeling, polyplex nanomicelle

## Abstract

Pathological remodeling after myocardial infarction (MI) involves multifactorial mechanisms, highlighting the need for combinatorial therapeutic strategies. Synthetic mRNAs offer design flexibility and represent a modality particularly suitable for such approaches. To investigate this, five genes (*Hgf*, *Igf1*, *Pdgfb*, *Cxcl12*, and *Tgfβ1*) were selected from a model in which extracellular vesicles secreted by human iPSC‐derived cardiomyocytes restored cardiac function. Synthetic mRNAs encoding these genes were delivered via polyplex nanomicelles by direct myocardial administration in mice with MI‐induced heart failure. Nanomicelles have been shown to provide stable encapsulation, enhanced local expression, and prolonged persistence. This treatment promoted angiogenesis via the PI3K–Akt–ETV4 axis, suppressed fibrosis via inhibition of the JNK/FOXO3 pathway, and enhanced repair by activating ERK signaling, together yielding multifaceted benefits, including tissue regeneration, improved contractility, and extended survival. These findings establish the therapeutic potential of multigene mRNA cocktail therapy for post‐MI heart failure and mark an important step toward developing new interventions for diseases characterized by complex remodeling.

## Graphical abstract

Five reparative factors identified during myocardial recovery after treatment with extracellular vesicles derived from human iPS cell‐derived cardiomyocytes were combined into an mRNA cocktail and delivered to infarcted hearts using polyplex nanomicelles. The treatment improved cardiac function and survival, highlighting the therapeutic potential of multigene mRNA therapy.

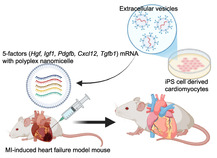

## 1 Introduction

Myocardial infarction (MI), the leading global cause of death, and its progression to ischemic cardiomyopathy together impose an escalating mortality burden, underscoring the urgent need for new therapeutic strategies [1, 2, 3]. Early reperfusion remains the only established intervention to limit infarct size. However, no therapy effectively targets the subsequent pathological remodeling, including ventricular dilation and contractile dysfunction [4, 5]. The mechanisms underlying this remodeling are multifactorial and involve inflammation, fibrosis, and other processes triggered by ischemic injury, making it unlikely that a single intervention can achieve meaningful benefits [6, 7, 8]. In this context, cell transplantation and extracellular vesicle (EV) administration, primarily acting via combinatorial paracrine effects, have been investigated [9, 10, 11, 12, 13]. However, the lack of standardization continues to hinder their clinical application.

Recent advances in mRNA technology have led to its transformation into a versatile platform for broadly applicable therapies. In ischemic heart disease, direct myocardial delivery of vascular endothelial growth factor (VEGF) mRNA has been shown to stimulate angiogenesis and improve cardiac function in animal models [14]. Clinical trials have confirmed its safety but revealed only modest functional gains [15], leading to discontinued development. We have previously demonstrated that EVs secreted by human induced pluripotent stem cell‐derived cardiomyocytes (hiPSC‐CMs) improve cardiac function in heart disease models [16]. In the treated myocardium, multiple genes showed rapid transient induction immediately after delivery. These findings suggest that the simultaneous delivery of multiple repair factors to the heart may act synergistically to enhance recovery.

Based on these findings, mRNA, a readily engineered modality suited for simultaneous delivery, was used to evaluate functional recovery in animal models of MI. Candidate genes were identified by profiling the reparative processes underlying the cardiac benefits of cell transplantation and EV therapy in ischemic heart disease, with an emphasis on those that were rapidly and transiently induced after treatment. Furthermore, to ensure safe myocardial delivery of these mRNAs, we employed a polyplex nanomicelle carrier. This carrier is a self‐assembled nanoparticle composed of block copolymers of polyethylene glycol (PEG) and polyamines, in which mRNA is stably encapsulated [17, 18]. Unlike lipid nanoparticles (LNPs), which are widely used for mRNA vaccines, nanomicelles have a diameter of several tens of nanometers and a nearly neutral surface charge surrounded by a dense PEG palisade [19]. They have been shown to enable local mRNA delivery without immune activation at the administration site. Their safety and efficiency in delivering mRNA have already been demonstrated not only in muscles but also in joints, the nervous system, and other organs, with their therapeutic efficacy confirmed in multiple disease and injury models [20, 21, 22, 23, 24, 25, 26, 27, 28].

Here, we propose a therapeutic concept that integrates the systematic analysis of tissue responses to ischemic stress with mRNA delivery of various candidate factors. This approach may enable next‐generation therapies to improve recovery from postinfarction heart failure.

## 2 Materials and Methods

### 2.1 Selection of Cardiac Reparative Factors

Clinical‐grade hiPSC‐CM sheets have demonstrated therapeutic efficacy in patients with severe ischemic heart disease. To investigate the underlying mechanisms, we harvested EVs from the culture medium of clinical‐grade hiPSC‐CMs by ultracentrifugation and administered them intramyocardially to a rat model of severe ischemic heart disease, resulting in improved cardiac function and survival [16]. Briefly, the clinical‐grade human iPSC line QHJI14s04 was established at the Center for iPS Cell Research and Application, Kyoto University (Kyoto, Japan), from peripheral blood mononuclear cells of a healthy donor homozygous for the human leukocyte antigen haplotype 17, the most frequent haplotype in the Japanese population. hiPSC‐CMs were cultured for 16 days, and the conditioned medium was collected, filtered to remove cellular debris, concentrated with denatured polyethylene glycol, and ultracentrifuged. The isolated EVs expressed representative exosomal markers, including CD63, syntenin, TSG101, and ALIX. MI was induced in 6‐week‐old nude rats (Charles River Laboratories Japan, Inc., Yokohama, Japan) by permanent ligation of the left coronary artery. 2 weeks later, rats with a reduced left ventricular ejection fraction (EF < 45%) were intramyocardially administered 1 × 10^10^ EV particles into the infarct border area. Subsequently, cardiac function improvement was confirmed. Myocardial tissue samples were collected immediately before EV administration and on days 3 and 7 afterward. Total RNA was isolated and sequenced at the NGS core facility of the Research Institute for Microbial Diseases, University of Osaka (Osaka, Japan). Candidate factors were selected from genes that showed strong transient upregulation on day 3.

### 2.2 Preparation of mRNAs

Template DNAs for in vitro transcription of mRNAs encoding TGFb, HGF, PDGFb, IGF1, or CXCL12 were prepared via PCR using PrimeSTAR Max DNA polymerase (Takara Bio). The nucleotide sequence of each construct was codon‐optimized as described in the Supplementary Text. In vitro transcription was performed using the template DNAs, IVTpro mRNA Synthesis System (Takara Bio), CleanCap Reagent AG (TriLink Biotechnologies), and N^1^‐methylpseudouridine‐5′‐triphosphate (TriLink Biotechnologies or FUJIFILM Wako Pure Chemical Corporation). Following transcription, DNase I was added to remove the template DNA. The resulting mRNAs were purified using RNAClean XP (Beckman Coulter) and dephosphorylated using Quick CIP (New England Biolabs). Dephosphorylated mRNA was further purified using the RNeasy Mini Kit (Qiagen) or Monarch RNA Cleanup Kit (New England Biolabs). The concentration of purified mRNAs was determined using the Qubit RNA Broad Range Assay Kit (Thermo Fisher Scientific) and Qubit 4 Fluorometer (Thermo Fisher Scientific). The lengths of the mRNAs were analyzed using an Agilent 2100 Bioanalyzer (Agilent Technologies) and the Agilent RNA 6000 nano Kit (Agilent Technologies). The mRNAs encoding Firefly luciferase (Luc) or Enhanced Green Fluorescent Protein (EGFP) were purchased from TriLink (San Diego, CA, USA).

### 2.3 Synthesis of Block Copolymer

A block copolymer, polyethylene glycol, Poly(N‐{N′‐(2‐aminoethyl)‐2‐aminoethyl}aspartamide) (PEG‐PAsp(DET)), was synthesized as previously reported [29]. Briefly, β‐Benzyl‐L‐aspartate N‐carboxyanhydride (BLA‐NCA: Chuo Kasei Co. Ltd., Japan) was polymerized from the terminal primary amino group of α‐methoxy‐ω‐amino poly(ethylene glycol) (PEG‐NH2: Nippon Oil and Fats, Japan) (Mw 43 kDa) to obtain PEG‐block‐poly(β‐benzyl‐L‐aspartate) (PEG‐b‐PBLA) via ring‐opening polymerization. Then, triethylenetetramine (DET; Wako Pure Chemical Industries, Ltd., Japan) was introduced into the side chain of PBLA by aminolysis to obtain PEG‐PAsp (DET). The degree of DET polymerization was calculated to be 63 using 1H NMR spectroscopy (JEOL EX300 spectrometer, JEOL, Japan).

### 2.4 Preparation of Polyplex Nanomicelles Loading mRNAs

To prepare mRNA‐loaded polyplex nanomicelles, mRNAs and PEG‐PAsp(DET) were separately dissolved in 10 mM HEPES buffer (pH 7.3) and mixed by adjusting the N/P ratio (the residual molar ratio of the polycations in the amino groups to the mRNA phosphate groups) to 3.0, following our previous studies on mRNA administration to skeletal muscle and neural tissues [22, 26]. This N/P ratio was selected because stoichiometric charged nanomicelles are stably formed when N/P ≥ 3 [30, 31]. The final mRNA concentration was adjusted to 500 µg/mL, regardless of mRNA type. The nanomicelle particle size was confirmed to be ~ 90 nm by dynamic light scattering (Zetasizer Nano ZS, Malvern Instruments Ltd., Worcestershire, UK) (Figure S1).

### 2.5 Western Blotting for Confirming the Protein Production From mRNAs encoding 5 Factors

HeLa cells were cultured in DMEM supplemented with 10% fetal bovine serum (FBS) and 1% penicillin‐streptomycin in a humidified incubator at 37°C with 5% CO_2_. The mRNAs encoding TGFb1, HGF, IGF1, CXCL12, or PDGFb were transfected using Lipofectamine MessengerMAX (Thermo Fisher Scientific) according to the manufacturer's protocol. After harvesting for 24 h, cell lysates were prepared by homogenization in RIPA buffer containing protease and phosphatase inhibitors. Protein concentrations were determined using the Micro BCA Protein Assay Kit (Thermo Fisher Scientific) before loading onto a Bolt 4%–12% Bis‐Tris Plus gel (Thermo Fisher Scientific) for electrophoresis and subsequent transfer to PVDF membranes. Membranes were blocked with 5% nonfat dry milk and incubated with primary antibodies against TGFb1 (21898‐1‐AP, Proteintech), HGF (sc‐374 422, Santa Cruz), IGF1 (sc‐518 040, Santa Cruz), CXCL12 (sc‐518 066, Santa Cruz), or PDGFb (Proteintech 83 214‐5‐RR) and loading controls GAPDH (D16H11, Cell Signaling Technology) and *α*‐tubulin (T6199, Sigma–Aldrich). After incubation with an HRP‐conjugated secondary antibody (W402B, Promega), bands were visualized by chemiluminescence. Membranes were washed three times with TBST between incubations.

### 2.6 Ethical Statement on Animal Experiments

All animal experiments were performed in accordance with the protocols approved by the Animal Experimentation Committee of The University of Osaka (approval number: 05‐030−010). Animal care and use complied with the Principles of Laboratory Animal Care (National Medical Research Council) and Guide for the Care and Use of Laboratory Animals (National Institutes of Health).

### 2.7 Intramyocardial Administration of mRNA into Normal Mouse Hearts

To investigate the in vivo kinetics of mRNA‐loaded nanomicelles after cardiac delivery, 10‐week‐old female C57BL/6J mice (18–20 g; Jackson Laboratory Japan Inc., Yokohama, Japan) were used. The mice were anesthetized with 3% isoflurane (Pfizer Inc., Tokyo, Japan), intubated with a 24G cannula (Terumo Corporation, Tokyo, Japan), and connected to a rodent ventilator (Shinano Seisakusho Ltd., Tokyo, Japan). Anesthesia was maintained using 2% isoflurane. During the procedure, the animals were placed supine on a 40°C heating pad (Natsume Seisakusho Co., Ltd., Tokyo, Japan) to prevent hypothermia. A left thoracotomy was performed at the fourth intercostal space, and 1 µg/10 µL of Luc mRNA‐loaded nanomicelles or naked Luc mRNA was administered into the left anterior ventricular wall using a 30 G, 8 mm, 3/10 mL syringe (Becton, Dickinson and Company, Franklin Lakes, NJ, USA). After chest closure, the mice were extubated once spontaneous respiration was confirmed and returned to their cages after stabilization.

### 2.8 Myocardial Infarction Model and Intramyocardial Administration of mRNA

MI was induced in 10‐week‐old female C57BL/6J mice (18–20 g; Jackson Laboratory Japan, Inc., Yokohama, Japan). Mice were anesthetized with 3% isoflurane (Pfizer Inc., Tokyo, Japan), intubated with a 24G cannula (Terumo Corporation, Tokyo, Japan), and ventilated using a small animal respirator (Shinano Seisakusho Ltd., Tokyo, Japan). Anesthesia was maintained using 2% isoflurane. During the procedure, the animals were placed supine on a 40°C heating pad (Natsume Seisakusho Co., Ltd., Tokyo, Japan) to prevent hypothermia. Left thoracotomy was performed at the fifth intercostal space, the pericardium was opened, and the left coronary artery was ligated at the level of the left atrial appendage using a 7–0 silk suture (Natsume Seisakusho Co., Ltd., Tokyo, Japan). Successful induction of MI was confirmed by pallor and severe hypokinesia of the anterior and lateral walls of the left ventricle. After hemostasis, the chest was closed, and the mice were extubated once spontaneous respiration was confirmed, and returned to their cages after stabilization.

1 week after MI induction, transthoracic echocardiography (GE Healthcare, Chicago, IL, USA) was performed to evaluate left ventricular function, and mice with a left ventricular ejection fraction (LVEF) of <50% were enrolled in the study. The mice were reanesthetized and intubated as described earlier in the text, and a left thoracotomy was performed at the fourth intercostal space. After removing the intrathoracic adhesions, the MI border area was exposed. The border area was identified as the interface between the reddish, thick, normally perfused myocardium and the pale, thinned infarct region. One µg of each mRNA‐loaded nanomicelle, naked mRNA, or phosphate‐buffered saline (PBS; Thermo Fisher Scientific Inc., Waltham, MA, USA) was administered into the anterolateral border area using a 30 G, 8 mm, 3/10 mL syringe (Becton, Dickinson and Company, Franklin Lakes, NJ, USA). A needle was inserted parallel to the border area at an angle of approximately 15° to a depth of approximately 3 mm into the myocardium. After confirming the absence of blood reflux to ensure that the needle tip was positioned within the myocardial wall rather than the ventricular cavity, a total of 10 µL of mRNA‐loaded nanomicelle solution was injected. To prevent reflux, the needle was withdrawn 30 s after injection, and the injection site was immediately compressed with a cotton swab. After hemostasis, the chest was closed, the mice were extubated upon recovery from spontaneous breathing, and returned to their cages after stabilization.

### 2.9 Longitudinal In Vivo Evaluation and Ex Vivo Analysis Using an In Vivo Imaging System

To assess the longitudinal expression of Luc mRNA‐loaded nanomicelles and naked Luc mRNA in the heart, mice received an intramyocardial administration of Luc mRNA‐loaded nanomicelles or naked Luc mRNA, followed by in vivo imaging system (IVIS), Lumina (PerkinElmer, Inc., Waltham, MA, USA). For imaging, the mice were anesthetized with 3% isoflurane (Pfizer Inc., Tokyo, Japan) for induction and maintained with 2% isoflurane. D‐luciferin (150 mg/kg; ab143655, Abcam, Waltham, MA, USA) was administered intraperitoneally, and imaging was performed 10 mins later. The first assessment was performed 4 h after administration, followed by daily imaging from days 1–14.

For ex vivo analysis of Luc expression in nontarget organs after intramyocardial administration, mice received intramyocardial administration of Luc mRNA‐loaded nanomicelles, naked Luc mRNA, or PBS as described earlier in the text and were sacrificed 24 h later under 3% isoflurane anesthesia. d‐Luciferin (150 mg/kg) was administered intraperitoneally, and after 10 min, the hearts, lungs, livers, spleens, kidneys, and ovaries were harvested. Concurrently, blood samples were obtained from either the tail vein or inferior vena cava to evaluate systemic immune responses and biochemical parameters. A 0.5 M EDTA stock solution was prepared by dissolving 1.86 g of EDTA.2Na (DOJINDO Laboratories, Kumamoto, Japan) in distilled water and adjusting the pH to 8.0. Prior to blood collection, 4 µL of the EDTA stock solution was added to each syringe as an anticoagulant. Subsequently, 400 µL of blood was collected, resulting in a final EDTA concentration of 5 mM. Immediately after collection, the samples were gently inverted to ensure thorough mixing and transferred to 0.6 mL microcentrifuge tubes. The samples were kept on ice and centrifuged at 3,000 × g for 10 min at 4°C. The resulting platelet‐poor plasma was collected and stored at −80°C until further analysis. After perfusion with PBS, the organs were immersed in D‐luciferin and imaged. Luc expression was evaluated by bioluminescence imaging and quantified as radiance (photons/s/cm^2^/sr). Bioluminescent signals were analyzed using the Living Image Software v4.3.1 (PerkinElmer, Waltham, MA, USA). Ex vivo bioluminescence values of each organ were normalized to those of the organs administered an equivalent volume of PBS.

### 2.10 Evaluation of Immunological Responses and Hepatic and Renal Function

Plasma levels of IFN‐γ, IL‐4, IL‐5, IL‐6, IL‐12p70, and TNF‐α were quantified using the Luminex xMAP INTELLIFLEX system (Luminex Corporation, Austin, TX) with the ProcartaPlex Mouse Essential Th1/Th2 Cytokine Panel (6‐plex) (Thermo Fisher Scientific, Waltham, MA), according to the manufacturer's instructions. Hepatic and renal function markers were analyzed using a DRI‐CHEM NX600 analyzer (Fujifilm, Tokyo, Japan) with dedicated dry chemistry slides according to the manufacturer's protocol. Liver function was evaluated by measuring serum aspartate aminotransferase (AST) and alanine aminotransferase (ALT). Renal function was assessed by measuring the serum creatinine (Cre).

### 2.11 Echocardiographic Assessment

Cardiac function was assessed by transthoracic echocardiography 1 week after MI (before mRNA administration) and 2 and 4 weeks after mRNA administration. The assessments were performed in a blinded manner by at least two independent investigators and repeated several times. Mice were anesthetized with 1%–2% isoflurane (Pfizer Inc., Tokyo, Japan) at a depth sufficient to prevent bradycardia. M‐mode images of the left ventricle were acquired in the short‐axis view at the papillary muscle level using an echocardiography system (Vivid I; GE Healthcare, Chicago, IL, USA). The anterior wall thickness, posterior wall thickness, left ventricular end‐diastolic and end‐systolic diameters (Dd and Ds), ejection fraction (EF), and fractional shortening (FS) were measured. Each parameter was measured at least five times, and the mean value was used for analysis. In mRNA administration experiments, only mice with EF < 50% at 1 week after MI were included, whereas those with EF ≥ 50% were excluded.

### 2.12 Histological Analysis

The excised hearts were rapidly perfused with PBS to remove blood, fixed in 4% paraformaldehyde, and subjected to sucrose substitution. Samples were then embedded in Optimal Cutting Temperature compound (Sakura Finetek, Torrance, CA, USA), rapidly frozen in liquid nitrogen, and stored at −80°C. Frozen blocks were sectioned at 10 µm thickness using a cryostat, mounted on glass slides, and subjected to immunohistochemical analysis. The following primary antibodies were used: EGFP (ab290, 1:1000 dilution; Abcam, Cambridge, MA, USA), cardiac troponin T (MS‐295‐P, 1:100 dilution; Thermo Fisher Scientific, Waltham, MA, USA), CD31 (ab187377, 1:100 dilution; Abcam, Cambridge, MA, USA), α‐smooth muscle actin (αSMA; M0851, 1:50 dilution; DAKO, Carpinteria, CA, USA), and vimentin (ab8978, 1:100 dilution; Abcam, Cambridge, MA, USA). Secondary antibodies included Alexa Fluor 488 goat antimouse IgG (H + L) (A11001, 1:400 dilution; Thermo Fisher Scientific, Waltham, MA, USA) and Alexa Fluor 555 goat antirabbit IgG (H + L) (A21428, 1:200 dilution; Thermo Fisher Scientific, Waltham, MA, USA). Nuclear counterstaining was performed with Hoechst 33 342 (EJ‐091, 1:100 dilution; Dojindo Laboratories). The overall tissue architecture and spatial distribution of EGFP‐positive cells were examined using a fluorescence microscope (BZ‐810; Keyence, Osaka, Japan), and specific EGFP‐expressing cell types were identified using a confocal microscope (FV10i; Olympus, Tokyo, Japan). For histological evaluation, excised organs were fixed in 10% formalin, embedded in paraffin, and sectioned at 2 µm thickness with a microtome. Tissue inflammation and injury were assessed using hematoxylin and eosin (H&E) staining. Myocardial fibrosis was analyzed by Picrosirius Red staining, and the fibrotic area was quantified as the ratio of the fibrotic area to the total left ventricular area using the MetaMorph software (v7.10.3; Molecular Devices, San Jose, CA, USA). Fibrosis at the border and remote areas was quantified as the ratio of the fibrotic area to the corresponding left ventricular area. Angiogenesis was assessed by immunohistochemical staining for CD31 (ab28364, 1:100 dilution; Abcam, Cambridge, MA, USA) with visualization by 3,3′‐diaminobenzidine (DAB) and counterstaining with hematoxylin. Apoptotic cells were detected using the TUNEL In Situ Apoptosis Detection Kit (MK500; Takara Bio Inc., Shiga, Japan), visualized by 3,3′‐diaminobenzidine (DAB), and counterstained with hematoxylin.

### 2.13 RNA Extraction and Sequencing

At 24 hr after mRNA therapy, myocardial tissue was harvested from the border area of the mice with post‐MI heart failure. The left ventricular border area was dissected into small pieces, sealed in polypropylene tubes containing RNAlater (Thermo Fisher Scientific, Waltham, MA, USA), and stored at −80°C. RNA extraction, library preparation, and sequencing were outsourced to the NGS Core Facility at the Bioinformatics Center of the University of Osaka (Osaka, Japan). Normalized read counts were generated according to standard protocols and the manufacturer's instructions. Briefly, total RNA was extracted using the miRNeasy Mini Kit (QIAGEN, Hilden, Germany), and RNA quality was assessed using a Bioanalyzer (Agilent Technologies, Santa Clara, CA, USA). RNA sequencing libraries were prepared using the TruSeq Stranded mRNA Library Prep Kit (Illumina, San Diego, CA, USA) and subjected to paired‐end 101 bp sequencing runs on a NovaSeq X Plus system (Illumina, San Diego, CA, USA).

### 2.14 RNA‐Seq Data Processing

RNA‐seq FASTQ files were trimmed and filtered using Trimmomatic (v0.39) [32] to retain reads with base quality ≥ Q30, followed by quality control and validation. Reads were aligned to a custom reference genome incorporating codon‐optimized five‐factor sequences into GRCm38 (GENCODE M11) with HISAT2 (v2.2.1) [33], achieving mapping rates ≥90% for all samples. Gene‐level quantification was performed using RSEM (v1.3.3) [34]. Transcripts per million (TPM) values were calculated from the endogenous gene reads per million mapped reads, whereas the TPM values for the five exogenous factors were computed directly from the read counts. Differentially expressed genes (DEGs) were identified using DESeq2 (v1.38.3) [35] with thresholds of, log2 FC| ≥ 1, *q* < 0.05, and *p* < 0.05. Heatmaps were generated from log2 FC (TPM + 1) values converted to z‐scores and visualized using ComplexHeatmap [36].

### 2.15 Reactome Gene Set Analysis and Gene Set Enrichment Analysis

Reactome gene set analysis (ReactomeGSA) was performed using the Reactome platform (https://reactome.org/gsa/home) [37]. Gene set enrichment analysis (GSEA) was conducted using GSEA (v4.3.2; https://www.gsea‐msigdb.org/gsea/index.jsp) [38] with 1,309 Reactome and 5 pathway interaction database (PID) gene sets. Significant gene sets were defined by, NES| ≥ 1.5 and FDR < 0.05.

### 2.16 Gene Network Analysis

Pathway‐ and network‐based analyses of DEGs were conducted using ReactomeFIPlugIn (v8.0.10) [39] in Cytoscape (v3.10.3) [40]. Hub genes were identified using the Enrichment Map (v3.5.0) [41]. Transcription factor prediction was performed using iRegulon (v1.3) [42], focusing on DEGs within gene sets related to RNA metabolism, the cell cycle, DNA replication, DNA repair, and metabolism.

### 2.17 RT‐qPCR

cDNA was synthesized by reverse transcription of 1 µg of total RNA using High‐Capacity RNA‐to‐cDNA Kit (Thermo Fisher Scientific, Waltham, MA, USA). RT‐qPCR was performed using PowerTrack SYBR Green Master Mix for qPCR (Thermo Fisher Scientific, Waltham, MA, USA) and QuantStudio 6 Pro Real‐Time PCR System (Thermo Fisher Scientific, Waltham, MA, USA) to quantify gene expression levels of *Src*, *Ets1*, *Grb2*, *Etv4*, *Mapk3*, *Hck*, *Fgr*, *Mmp9*, *Smad2*, *Smad3*, *Fos*, and *Jun*. Target gene expression levels were normalized to those of *Gapdh*. Primer sequences are presented in the Supplementary Table.

### 2.18 Statistical Analysis

Descriptive data are presented as mean ± standard error (SEM). Comparisons between two independent groups were performed using an unpaired Student's t‐test. For comparisons among three or more groups, one‐way analysis of variance (ANOVA) with the Tukey–Kramer posthoc test was applied. Cumulative survival rates were estimated using the Kaplan–Meier method and compared using the log‐rank test. Longitudinal radiance data from IVIS imaging and serial echocardiographic measurements were analyzed using a repeated‐measures mixed‐effects model, including group, time, and their interaction as fixed effects, followed by Tukey–Kramer posthoc testing. All analyses were performed using GraphPad Prism v10 software (GraphPad Software, San Diego, CA, USA). RNA‐seq data were analyzed using the R software (v4.4.3; http://www.r‐project.org). Normality was tested using the Shapiro–Wilk test and homogeneity of variance using Bartlett's test. For normally distributed data, the Student's t‐test was applied; for non‐normally distributed data, the Mann–Whitney U test was used. Statistical significance was set at *p* < 0.05.

## 3 Results

### 3.1 Selection of Cardiac Reparative Factors From hiPSC‐CM–Derived EVs

**FIGURE 1:**
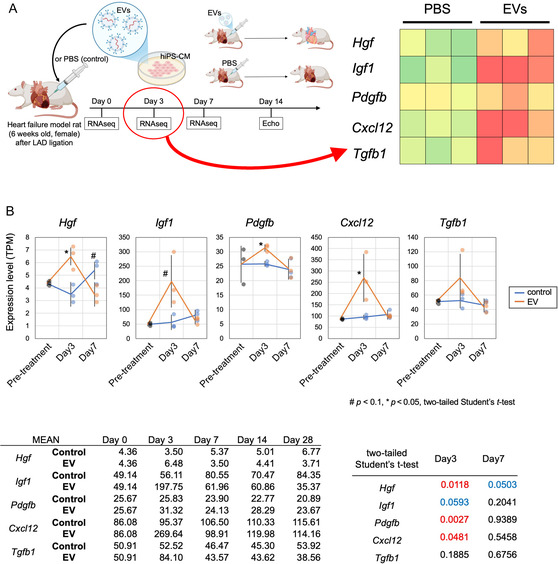
Selection of cardiac reparative factors from hiPSC‐CM–derived EV therapy. (A) Experimental design of clinical‐grade hiPSC‐CM‐derived EV therapy in a post‐MI heart failure model. 2 weeks after infarction, EVs were administered intramyocardially into the infarct border area of mice with impaired cardiac function 2 weeks after infarction. Myocardial tissues were collected on days 0, 3, and 7 for RNA sequencing. Echocardiography on day 14 showed a significant improvement in cardiac function in the EV‐treated group compared to the controls. The heatmap displays the expression of the five selected factors identified by RNA sequencing on day 3. (B) Transient expression profiles of the five genes in myocardial tissue from the infarct border area before treatment and on days 3 and 7 after treatment. Data are shown as mean ± SEM. Sample size: *n* = 4 per group at each time point. hiPSC‐CM, human induced pluripotent stem cell‐derived cardiomyocyte; EV, extracellular vesicle; LAD, left anterior descending artery; MI, myocardial infarction; Echo, echocardiography; RNA‐seq, RNA sequencing.

A previously established model of intramyocardial administration of hiPSC‐CM EVs in rats with post‐MI heart failure demonstrated improved cardiac function and survival on day 14 [16]. RNA‐seq of myocardial tissue on days 0, 3, and 7 identified a subset of genes that were transiently upregulated on day 3 and downregulated on day 7 in EV‐treated hearts. Five genes—*Hgf*, *Igf1*, *Pdgfb*, *Cxcl12*, and *Tgfb1*—were selected as cardiac repair factors based on prior evidence of their roles in functional recovery [8, 16, 43, 44, 45, 46, 47, 48, 49, 50, 51, 52, 53, 54] (Figure 1A,B). In brief, HGF promotes cytoprotection and angiogenesis after tissue injury, IGF1 supports cardiomyocyte metabolism and contractile function, PDGF‐B contributes to immune cell recruitment and stabilization of newly formed vessels, CXCL12 facilitates recruitment of hematopoietic stem and progenitor cells to injured myocardium, and TGFβ1 suppresses excessive immune responses and exerts anti‐inflammatory effects that promote tissue repair. Although chronic activation of TGFβ1 has been shown to promote pathological fibrosis and adverse cardiac remodeling, transient expression during the acute phase of injury can exert anti‐inflammatory and tissue‐reparative effects [55, 56]. Notably, the temporal pattern of these gene expression changes closely paralleled the expected duration of the mRNA‐driven protein expression. Therefore, we hypothesized that the simultaneous mRNA expression of these genes could recapitulate the key reparative signaling pathways associated with cell therapy–mediated cardiac recovery. Synthetic mRNAs encoding these factors were generated by IVT, and robust protein expression was validated by Western blotting (Figure S2). These findings establish HGF, IGF1, PDGFB, CXCL12, and TGFB1 as candidate reparative factors for mRNA‐based therapies.

### 3.2 Expression Dynamics After Intramyocardial Administration Assessed by IVIS

**FIGURE 2:**
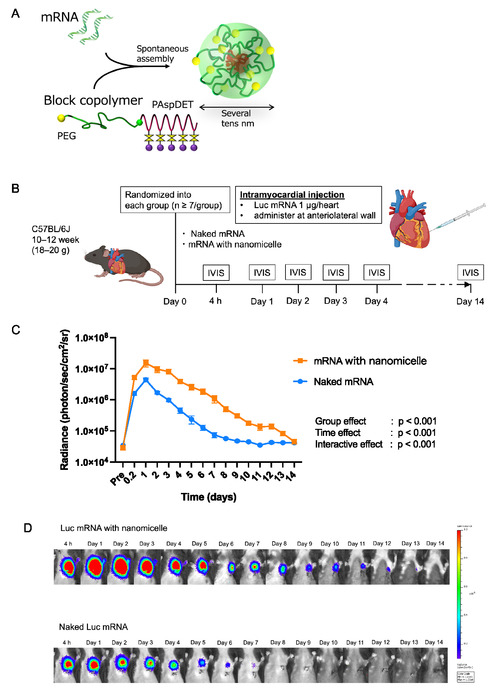
Serial in vivo protein expression after intramyocardial administration of mRNA‐loaded polyplex nanomicelles (A) Schematic representation of mRNA‐loaded polyplex nanomicelle preparation. (B) Experimental design: Mice received intramyocardial administration of Luc mRNA–loaded nanomicelles or naked Luc mRNA, followed by serial IVIS imaging for 14 days (*n* = 7–10 per group). The experiments were independently repeated twice. (C) Serial changes in mean radiance measured using IVIS. Data are shown as mean ± SEM. Radiance was analyzed using a mixed‐effects model with group, time, and interaction terms. (D) Representative IVIS images at the indicated time points. Luc, firefly luciferase; mRNA, messenger RNA; IVIS, in vivo imaging system; SEM, standard error of the mean.

Polyplex nanomicelles (Figure 2A) were evaluated for their ability to enhance the magnitude and duration of cardiac mRNA expression. Luc mRNA–loaded nanomicelles or an equivalent dose of naked Luc mRNA was intramyocardially administered to mouse hearts (*n* = 7–10 per group), followed by serial IVIS imaging from 4 h to day 14 (Figure 2B). Both groups exhibited detectable Luc expression at 4 h, which peaked on day one and remained elevated for approximately 3 days. mRNA‐loaded nanomicelles showed significantly higher expression than naked mRNA at all time points, declining to ~10% of the peak by day 7 yet persisting until day 13. In contrast, naked mRNA expression diminished by day 7 (Figure 2C,D, and Figure S3). Nanomicelle encapsulation ensured prolonged and enhanced cardiac mRNA expression.

### 3.3 Ex Vivo Biodistribution and Cellular Uptake Analysis

**FIGURE 3:**
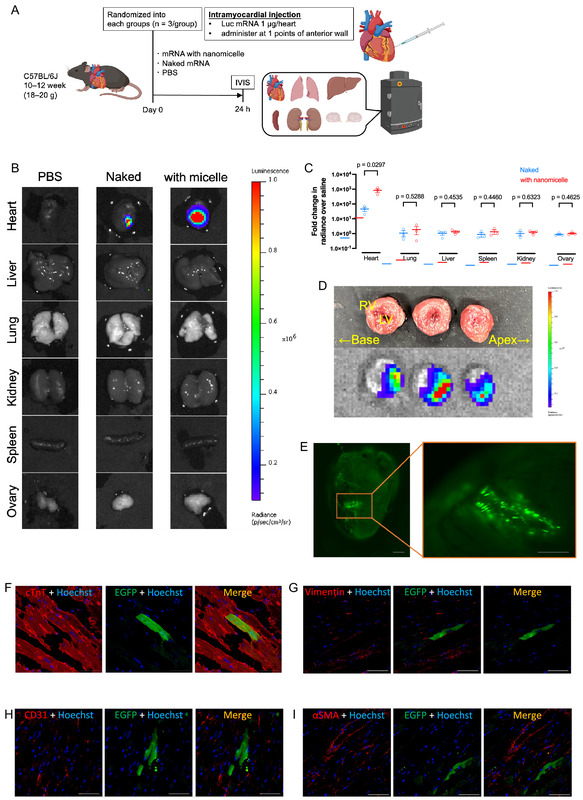
Ex vivo analysis after intramyocardial administration of mRNA‐loaded nanomicelles (A) Experimental design: Normal mice received intramyocardial administration of Luc mRNA–loaded nanomicelles, naked Luc mRNA, or PBS, followed by ex vivo analysis at 24 h (*n* = 3 per group). The experiments were independently repeated twice. (B) Ex vivo IVIS imaging of Luc expression in excised organs. (C) Quantification of Luc expression in organs normalized to PBS‐injected controls (*n* = 3 per group). Data shown as mean ± SEM. Statistical analyses were performed using the unpaired t‐test. (D) IVIS image of a short‐axis section of the heart 24 h after Luc mRNA–loaded nanomicelle administration. (E) Whole‐heart image 24 h after EGFP mRNA–loaded nanomicelle administration, showing EGFP expression at the injection site (scale bar, 1,000 μm) and a magnified view of the expression area (scale bar, 500 μm). (F–I) Immunofluorescence of heart sections 24 h after EGFP mRNA–loaded nanomicelle administration, showing EGFP (green) in cardiomyocytes (cTnT, red; (F)), fibroblasts (vimentin, red; (G)), endothelial cells (CD31, red; (H)), and smooth muscle cells (αSMA, red; (I)). Nuclei were counterstained with Hoechst (blue). Scale bars, 50 μm. Luc, firefly luciferase; mRNA, messenger RNA; PBS, phosphate‐buffered saline; IVIS, in vivo imaging system; SEM, standard error of the mean; EGFP, enhanced green fluorescent protein; RV, right ventricle; LV, left ventricle; cTnT, cardiac troponin T; αSMA, α‐smooth muscle actin.

To evaluate expression in nontarget organs, Luc mRNA–loaded nanomicelles or an equivalent dose of naked mRNA was intramyocardially administered, and major organs (heart, lung, liver, kidney, spleen, and ovary) were harvested 24 h later for ex vivo IVIS analysis (*n* = 3 per group) (Figure 3A). Nanomicelles yielded higher expression at the cardiac administration site than the naked mRNA, with minimal expression in nontarget organs (Figure 3B). Normalization to PBS controls confirmed significantly higher protein expression in the heart with nanomicelles, whereas nontarget organs showed negligible protein expression (Figure 3C). In the mRNA‐loaded nanomicelle group, no inflammatory cell infiltration or histological tissue injury was observed in the harvested organs, including the heart (Figure S4). Similarly, no elevation of inflammatory cytokines or abnormalities in hepatic or renal function was detected (Figure S5). Cross‐sectional IVIS revealed localized Luc expression at the administration site (Figure 3D). To define the cellular source of expression, EGFP mRNA–loaded nanomicelles were intramyocardially administered, and hearts were harvested after 24 h for immunohistochemistry. Whole‐heart fluorescence imaging revealed a strong EGFP expression at the anterior wall administration site (Figure 3E). Immunostaining revealed EGFP expression in cardiomyocytes identified by cardiac troponin T (Figure 3F) but not in fibroblasts (Vimentin, Figure 3G), endothelial cells (CD31, Figure 3H), or smooth muscle cells (αSMA, Figure 3I). Nanomicelle encapsulation ensures cardiomyocyte‐specific expression while suppressing off‐target expression in noncardiac organs.

### 3.4 Therapeutic Efficacy of Five‐Factor mRNA‐Loaded Nanomicelles

**FIGURE 4:**
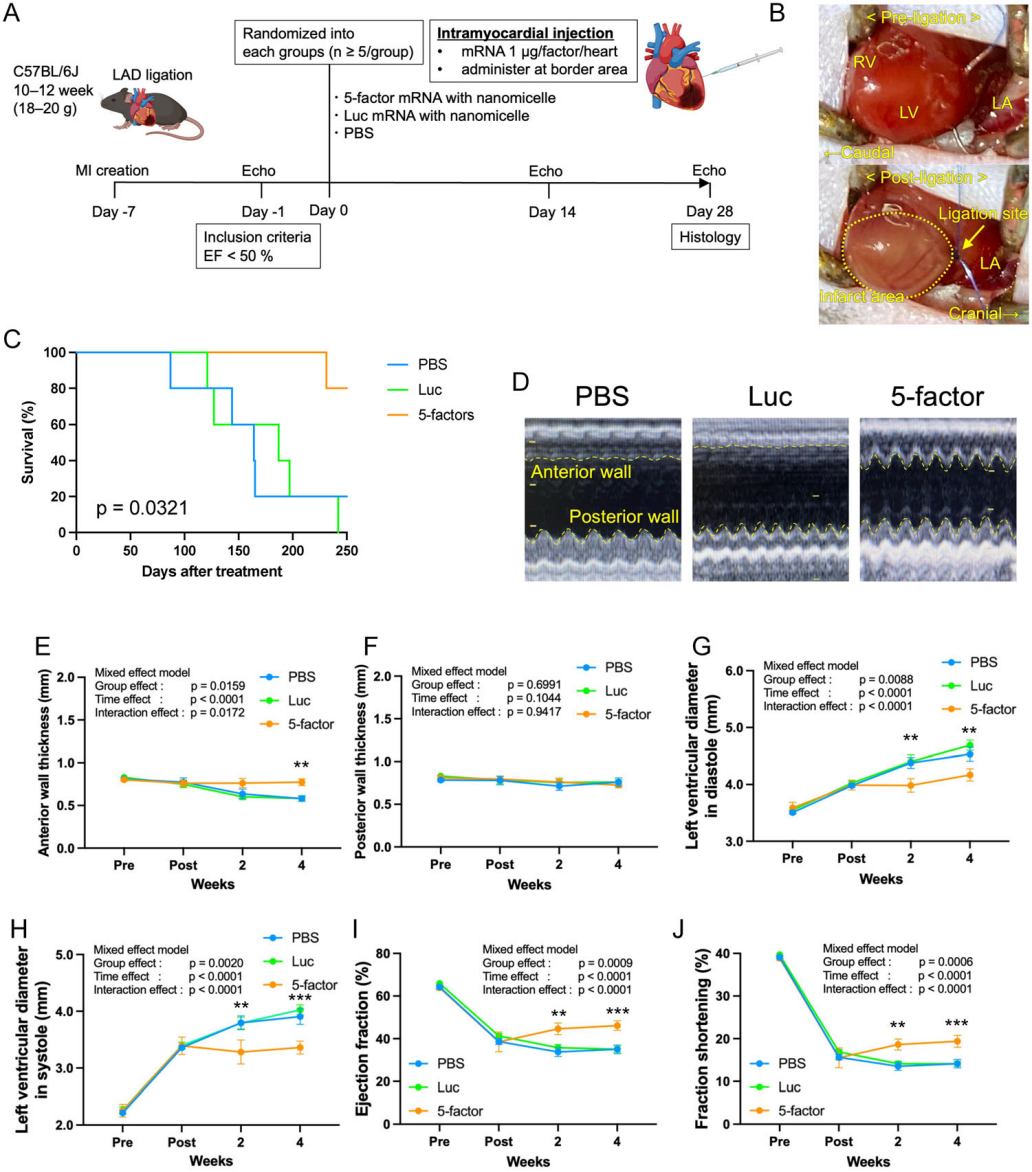
Cardiac functional assessment by five‐factor mRNA‐loaded nanomicelles. (A) Experimental design to evaluate the therapeutic effect of five‐factor mRNA–loaded nanomicelles in a post‐MI heart failure model. Control groups received Luc mRNA–loaded nanomicelles or PBS (*n* = 5–8 per group). (B) MI was induced by left thoracotomy and ligation of the LAD after intubation and exposure of the heart. Successful infarction was confirmed by pallor and severe asynergy of the LV myocardium in the coronary perfusion area. (C) Kaplan–Meier survival curves (*n* ≥ 5 per group), analyzed by log‐rank test. (D) Representative LV M‐mode echocardiographic images at 4 weeks. (E–J) Longitudinal changes in anterior wall thickness (E), posterior wall thickness (F), LV end‐diastolic diameter (G), LV end‐systolic diameter (H), LV ejection fraction (I), and fractional shortening (J) analyzed by transthoracic echocardiography in the five‐factor group versus controls (*n* = 5–8 per group). Functional assessment was performed at baseline (Pre), 1 week after MI prior to RNA treatment (Post), and 2 and 4 weeks after treatment. Data are shown as mean ± SEM. Longitudinal LV parameters were analyzed by a mixed‐effects model including group, time, and group × time interaction, followed by Tukey–Kramer posthoc testing. *p < 0.05, **p < 0.01, **p < 0.001 for five‐factor vs. Luc. LAD, left anterior descending artery; mRNA, messenger RNA; Luc, firefly luciferase; PBS, phosphate‐buffered saline; MI, myocardial infarction; EF, ejection fraction; Echo, echocardiography; LV, left ventricular; LA, left atrium; RV, right ventricle; SEM, standard error of the mean.

To investigate the therapeutic potential of multifactor mRNA therapy, five‐factor mRNA–loaded nanomicelles were intramyocardially administered to mice with post‐MI heart failure, and cardiac function was evaluated by echocardiography at two and 4 weeks, with long‐term survival analysis up to 250 days (Figure 4A,B). The five‐factor treatment significantly improved survival compared to Luc mRNA–loaded nanomicelles and PBS controls (Figure 4C). At 4 weeks, representative M‐mode images demonstrated persistent anterior wall akinesia in the control group, whereas wall motion was restored in the five‐factor group (Figure 4D). Quantitative analysis showed attenuation of the anterior wall thinning at 2 weeks, which was sustained at 4 weeks (Figure 4E), with no significant changes in the posterior wall thickness across groups (Figure 4F). Five‐factor nanomicelles also reduced pathological left ventricular dilation in both systole and diastole (Figure 4G,H) and improved left ventricular ejection fraction and fractional shortening relative to controls (Figure 4I,J). Five‐factor mRNA–loaded nanomicelles improve cardiac function and survival after MI.

### 3.5 Histological Analysis

**FIGURE 5:**
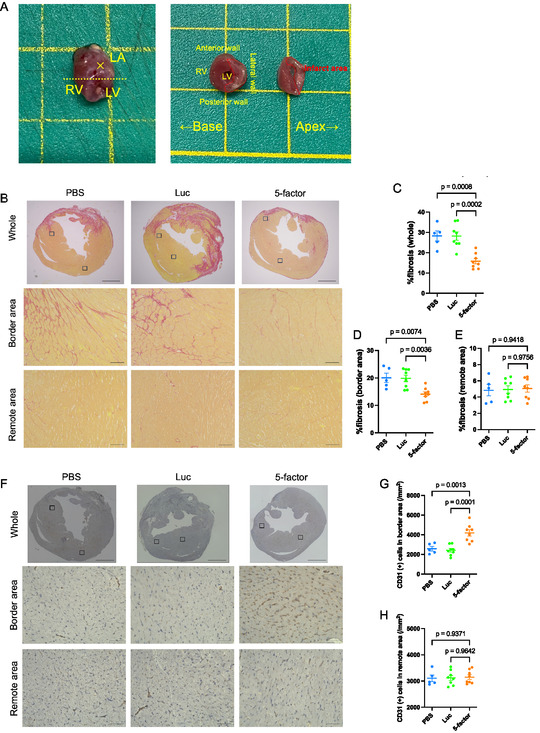
Histological analysis. (A) Macroscopic appearance of excised hearts from the PBS group. The hearts were perfused with PBS, sectioned transversely into the mid–left ventricle, fixed, and processed for histological analysis. The infarct area is outlined by a red dotted line; the border area is defined as the interface between the infarct and adjacent viable myocardium for subsequent analyses. (B) Picrosirius Red staining at 4 weeks after treatment showing the infarct and fibrotic areas in each group. Representative whole‐section images and magnified views of the border and remote areas are shown (whole‐image scale bar, 1,000 μm; magnified image scale bar, 100 m). (C–E) Quantification of fibrosis in the entire left ventricle (C), border area (D), and remote area (E) (*n* = 5–8 per group). (F) CD31 immunostaining of hearts harvested 4 weeks after treatment to evaluate microvascular density. Representative whole‐section images and magnified views of the border and remote areas are shown (whole‐image scale bar, 1,000 μm; magnified image scale bar, 100 m). (G,H) Quantification of microvascular density at the border (G) and remote areas (H) (*n* = 5–8 per group). Data are shown as mean ± SEM. Statistical analysis was performed using one‐way ANOVA with Tukey–Kramer posthoc testing. LA, left artium; LV, left ventricle; RV, right ventricle; PBS, phosphate‐buffered saline; Luc, firefly luciferase; SEM, standard error of the mean; ANOVA, analysis of variance.

The hearts harvested 4 weeks after treatment were sectioned at the mid‐ventricular short axis (Figure 5A). The border area was defined as the interface between the infarcted and the viable myocardium. Sirius Red staining revealed smaller infarcts in the five‐factor mRNA–loaded nanomicelle group than in the Luc mRNA–loaded nanomicelles or PBS controls (Figure 5B,C), with fibrosis markedly suppressed in the border area (Figure 5B,D). No differences were observed in remote areas (Figure 5B,E). CD31 staining demonstrated increased vessel density in the border area of the five‐factor group compared to that in the controls (Figure 5F,G), with no changes in the remote area (Figure 5F,H). Moreover, the number of TUNEL‐positive cells was significantly reduced in the mRNA‐loaded nanomicelle group compared with the control group, indicating suppression of cardiomyocyte apoptosis (Figure S6). These findings indicated that the five‐factor mRNA–loaded nanomicelles promoted angiogenesis, suppressed fibrosis, limited infarct expansion, and promoted cardiomyocyte survival after MI.

### 3.6 Five‐Factor Treatment Remodels Transcriptomes through Five Hallmark Pathways

**FIGURE 6:**
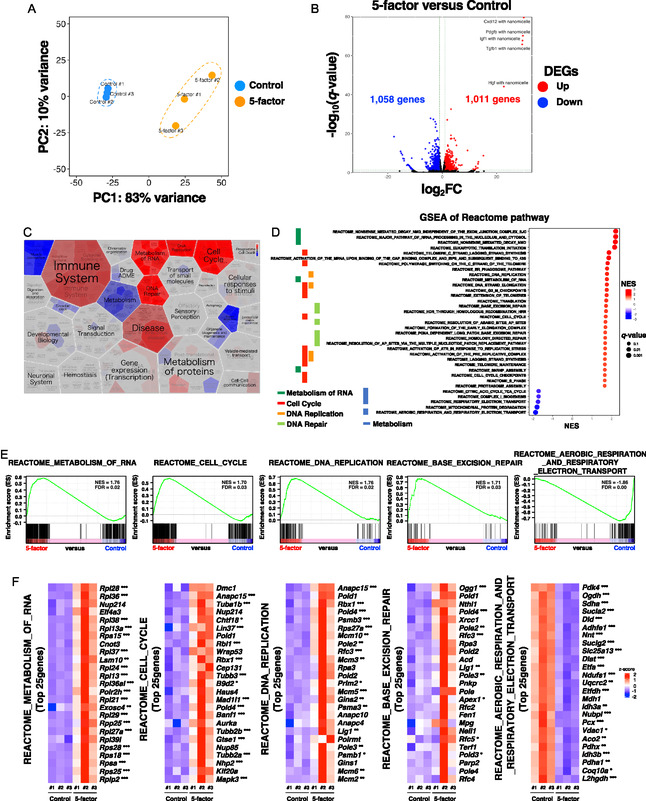
Transcriptome alterations induced by intramyocardial administration of five‐factor mRNAs using nanomicelles (A) Principal component analysis (PCA) plot of transcriptome data from five‐factor–treated hearts. (B) Volcano plot of differentially expressed genes (DEGs) between the five factors and control groups. Upregulated genes (log_2_FC ≥ 1, q < 0.05) and downregulated genes (log_2_FC ≤ –1, q < 0.05) are indicated in red and blue, respectively. (C) Results of the comparative pathway analysis of RNA‐seq data using ReactomeGSA. (D) Gene Set Enrichment Analysis (GSEA) performed using Reactome gene sets. Among the 1,309 Reactome gene sets, 35 were enriched in each group. NES, normalized enrichment score. (E) Representative enrichment plots of REACTOMEMETABOLISMOFRNA, CELLCYCLE, DNAREPLICATION, BASEEXCISIONREPAIR, and AEROBICRESPIRATIONANDRESPIRATORYELECTRONTRANSPORT gene sets. (F) Heatmap of the top 25 genes from each gene set showing the z‐scores of log_2_FC [TPM + 1] from the RNA‐seq data. *q < 0.05, **q < 0.01, ***q < 0.001 indicate significant differences by DESeq2 comparing the five‐factor group to controls. PCA, principal component analysis; DEG, differentially expressed gene; RNA‐seq, RNA sequencing; GSEA, Gene Set Enrichment Analysis; NES, normalized enrichment score; TPM, transcripts per million; FC, fold change.

To elucidate the molecular mechanisms underlying the improvement of MI by the five‐factor therapy, RNA‐seq analysis was performed. Principal component analysis (PCA) of the transcriptomic profiles revealed a clear separation between the control and five‐factor groups, indicating distinct global gene expression patterns (Figure 6A). Differential expression analysis using DESeq2 identified 1,011 upregulated and 1,058 downregulated genes in the five‐factor group compared to the controls (Figure 6B). The five‐factor mRNAs delivered in the nanomicelles were codon‐optimized and distinguishable from endogenous transcripts. These exogenous mRNAs were ranked among the top five expressed genes and were markedly upregulated relative to controls (Figure 6B and Figure S7A). In contrast, the endogenous counterparts exhibited a trend toward increased expression of some genes (Figure S7B). Pathway‐level analyses were performed using ReactomeGSA and GSEA software. ReactomeGSA revealed that ribosomal RNA‐associated metabolism of RNA (*p* = 1.4 × 10^−5^), cell cycle (*p* = 6.3 × 10^−4^), DNA replication (*p* = 1.7 × 10^−5^), and DNA repair (*p* = 1.5 × 10^−3^) were significantly activated in the five‐factor group, whereas mitochondria‐related metabolism (*p* = 3.5 × 10^−2^) was enriched in the control group (Figure 6C). Notably, many of the enriched gene sets converged into five representative categories within ReactomeGSA (Figure 6D,E). GSEA further supported these findings, showing enrichment of the top 25 genes across these pathways, many of which were significantly altered in DESeq2 (Figure 6F). Taken together, these results define five representative pathways—metabolism of RNA, cell cycle, DNA replication, DNA repair, and mitochondrial metabolism—as the principal transcriptomic alterations induced by 5‐factor treatment. The coordinated regulation of these pathways is likely to play a central role in the reparative effects of five‐factor therapy and may underlie the observed improvement in MI.

### 3.7 Five Key Pathway Changes Associated with Five‐Factor Treatment Involve ERK, JNK, and PI3K Signaling

**FIGURE 7:**
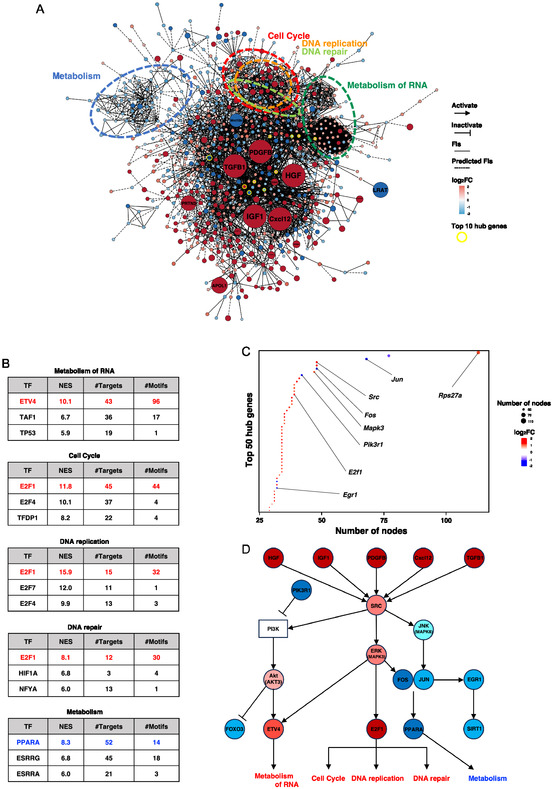
Gene network analysis of five‐factor mRNA–loaded nanomicelles (A) Network visualization of differentially expressed genes (DEGs) using reactome functional interactions (FI). The largest subnetworks, protein‐protein interactions, and gene regulatory networks were identified among the 2,069 DEGs. Genes are colored by log_2_ fold change (log_2_FC). Dotted circles indicate the positions of key pathway genes, including METABOLISM_OF_RNA, CELL_CYCLE, DNA_REPLICATION, DNA_REPAIR, and METABOLISM. (B) Prediction of transcription factor‐regulated genes in each pathway. The top three transcription factors associated with each set were presented. (C) Visualization of the top 50 hub genes within the network. (D) Schematic model illustrating changes in gene expression in response to five‐factor mRNA loaded with five‐factor mRNAs. DEG, differentially expressed gene; FI, Functional Interaction; log_2_FC, log_2_ fold change.

Gene network analysis was performed to elucidate the molecular alterations underlying these five characteristic pathways. The force‐directed layout revealed distinct clusters corresponding to the five gene sets, each of which is highlighted with dotted circles (Figure 7A). Transcriptional regulator analysis identified several key factors: ETV4 linked to RNA metabolism; E2F1 associated with the cell cycle, DNA replication, and DNA repair; and PPARA related to metabolic pathways (Figure 7B). Hub genes, defined by extensive overlapping interactions, have also been characterized. The top 50 hub genes included Src, associated with five‐factor signaling; Mapk3 and Fos, related to ERK; Jun, linked to JNK; and Pik3r1, a negative regulator of PI3K (Figure 7C and Figure S7C). Interestingly, changes in the expression of these genes were limited when a single factor was administered; however, the effect increased dramatically when all five factors were administered together. These results highlight a central node within the regulatory network that may play a key role in the changes in gene expression observed following the administration of all five factors (Figure S8). These findings highlight the central nodes within the regulatory networks. An integrated model was constructed to summarize the molecular effects of the five‐factor treatments (Figure 7D). In this model, ERK and PI3K are activated, RNA metabolism is stimulated via ETV4, and the cell cycle, DNA replication, and DNA repair processes are activated by E2F1. Activation of the PPARα–Sirt1 complex, previously reported to repress mitochondrial gene expression and contribute to heart failure pathogenesis [57], was attenuated. Specifically, Ppara and Sirt1 were elevated in MI controls but were suppressed following five‐factor treatment, suggesting that inhibition of JNK signaling contributes to this effect. Collectively, these findings demonstrate that the five‐factor treatment orchestrates coordinated remodeling of the ERK, PI3K, and JNK pathways, thereby promoting reparative processes and suppressing maladaptive signaling in MI.

## 4 Discussion

This is the first in vivo study to demonstrate that a multigene mRNA cocktail can ameliorate postinfarction remodeling. RNA‐seq analysis revealed activation of the MEK–ERK–ETV4/E2F1 and PI3K–Akt–ETV4 axes, together with suppression of the maladaptive JNK–AP1–PPARA axis. These coordinated signaling changes align with the core mechanisms of pathological remodeling, including cardiomyocyte survival, angiogenesis, and fibrosis regulation, and are consistent with the observed improvements in vascularization, fibrosis suppression, cardiac function, and survival, highlighting the therapeutic promise of multigene mRNA therapy in ischemic heart failure.

Although protein expression following a single administration of mRNA‐loaded nanomicelles persisted for approximately 2 weeks, improvement in cardiac function was maintained for at least 4 weeks after treatment. Consistent with previous studies showing that transient mRNA expression can induce sustained functional benefits after MI [58, 59], these findings suggest that mRNA therapy may act by transiently activating early reparative responses—including inflammatory modulation, suppression of cardiomyocyte death, and promotion of angiogenesis—that subsequently mitigate adverse left ventricular remodeling. Thus, early intervention with transient mRNA‐driven protein expression may redirect the myocardium toward functional recovery and produce durable improvements in cardiac function.

The activation of the PI3K–Akt–ETV4 axis represents a central mechanism that couples angiogenesis with cardioprotection. The PI3K–Akt pathway is recognized as a key regulator of postinfarction repair, engaging downstream effectors including eNOS, VEGF, mTOR, and GSK‐3β to drive cell proliferation, angiogenesis, and cardiomyocyte survival [60, 61, 62]. Importantly, five‐factor treatment also activated the transcription factor ETV4, which is known to regulate RNA metabolism and proangiogenic gene programs [63, 64, 65]. This coordinated PI3K–Akt–ETV4 activation plays a dual role in promoting angiogenesis and enhancing RNA metabolism. Consistent with these molecular changes, histological analysis revealed an increase in CD31‐positive vessels in the infarct border area, which directly linked pathway activation to structural improvements.

FOXO3 is a critical downstream effector of the PI3K–Akt pathway and exacerbates postinfarction remodeling when upregulated [66, 67, 68]. As a transcription factor, FOXO3 drives apoptosis and fibrosis, whereas its inhibition confers cardioprotection [67, 68]. In this study, Foxo3 expression was markedly increased in controls but was suppressed by the five‐factor treatment through Akt signaling. Concordantly, histological analysis demonstrated a pronounced reduction in fibrosis in the infarct border area, directly linking FOXO3 suppression to fibrosis attenuation, preservation of ventricular structure, and maintenance of contractile performance.

The MAPK cascade comprises four branches—ERK, JNK, p38, and ERK5—that govern proliferation and differentiation across cardiomyocytes, fibroblasts, endothelial cells, and macrophages [69, 70, 71]. Among these, the ERK pathway has been implicated in myocardial repair and survival [72], and a five‐factor treatment induces its activation. Of particular note was the induction of the transcription factor E2F1, a master regulator of cell‐cycle progression, DNA replication, and DNA repair [73, 74]. RNA‐seq analysis revealed the enrichment of E2F1‐driven programs, indicating the reactivation of transcriptional networks fundamental to myocardial regeneration.

Suppression of the JNK pathway and modulation of the PPARα–Sirt1 axis constituted additional key findings. The JNK cascade promotes cardiomyocyte apoptosis, inflammation, and fibrosis [75, 76, 77], and RNA‐Seq analysis confirmed its inhibition. PPARα, a nuclear receptor regulating lipid metabolism and mitochondrial function, triggers metabolic derangements in heart failure when excessively activated [78]. In the control hearts, Ppara and Sirt1 expression levels were elevated, whereas the five‐factor treatment suppressed their induction. These results indicate that five‐factor therapy attenuated maladaptive activation of the PPARα–Sirt1 axis through JNK inhibition, thereby preserving mitochondrial homeostasis and stabilizing energy metabolism. This mechanism likely contributes to the structural preservation and functional recovery of the failing heart.

Collectively, these findings establish that the PI3K–Akt–ETV4/FOXO3, ERK–E2F1, and JNK–PPARα–Sirt1 axes converge through reciprocal crosstalk to promote cardiomyocyte survival, angiogenesis, fibrosis control, and metabolic stabilization. Akt enhanced angiogenesis and survival, ERK–E2F1 activated reparative pathways, JNK inhibition mitigated fibrosis, and regulation of the PPARα–Sirt1 axis stabilized metabolism. The integrated activity of these pathways curtails pathological remodeling, yielding durable improvements in cardiac function and survival after MI.

The delivery system characteristics are central to the success of this approach. Recent comprehensive analyses of LNP–mRNA technologies have highlighted how advances in chemical design, formulation strategies, and clinical development have expanded the therapeutic applicability of mRNA‐based medicines [79]. However, LNP‐induced inflammation can significantly reduce the efficacy of mRNA therapeutics [80]. LNPs induce potent immune responses through adjuvant‐like effects in vaccine settings, yet this same property may constrain their use in therapeutic contexts such as cardiac repair, where excessive inflammation is detrimental [81]. In particular, localized tissue damage and the recruitment of inflammatory cells may counteract the regenerative signals intended by the encoded proteins. Although extracellular vesicles (EVs) derived from stem or progenitor cells have attracted considerable interest as therapeutic agents for cardiovascular diseases, their clinical application remains challenged by cargo heterogeneity and their complex mechanisms of action [82]. EV‐based and synthetic nanoparticle delivery systems are characterized by a tradeoff between low immunogenicity and efficient delivery, including endosomal escape [83]. Polyplex nanomicelles represent a delivery strategy designed to balance these competing constraints. The nanomicelles, previously demonstrated to induce strong local mRNA expression with limited immune activation in the skeletal muscle, articular cartilage, brain, and spinal cord [18, 20, 21, 22, 23, 24, 25, 26, 27, 28], have not been investigated in the heart. Here, the nanomicelles provided stable encapsulation and enhanced and prolonged cardiac expression. In contrast to lipid nanoparticles, which show substantial off‐target accumulation in the liver, spleen, and lungs even after direct cardiac administration [84], nanomicelles restrict expression primarily to cardiomyocytes at the site of administration.

Recent advances in targeted drug delivery systems have also enabled more precise therapeutic strategies; for example, siRNA delivery using macrophage membrane–fused liposomes has been reported to spatiotemporally modulate the CCL2–CCR2 axis [85]. Additionally, lipid‐based intelligent delivery vehicles capable of responding to physical and physiological activation have recently been proposed as strategies to improve the spatiotemporal control of therapeutic delivery [86]. These delivery properties ensured efficient multigene expression in the heart, underpinning the observed functional and survival benefits.

## 5 Conclusions

This study provides the first in vivo evidence that a multigene expression strategy using mRNA can effectively regulate postinfarction remodeling in the heart. Coordinated activation of the PI3K–Akt–ETV4 axis to promote angiogenesis, suppression of the JNK/FOXO3 pathway to limit fibrosis, and activation of the ERK pathway to enhance repair collectively drive tissue regeneration, improve cardiac function, and prolong survival. The properties of the polyplex nanomicelles serve as a critical foundation for this strategy, enabling efficient delivery and sustained expression. These findings highlight the therapeutic potential of mRNA cocktail therapy in the complex pathological setting of postinfarction heart failure and represent a significant step toward new treatment options.

## Author Contributions

**Kazuma Handa**: conceptualization, data curation, formal analysis, funding acquisition, investigation, methodology, project administration, validation, visualization, writing – original draft, writing – review, and editing. **Takuji Kawamura**: conceptualization, funding acquisition, supervision, writing – review, and editing. **Yasunobu Mano**: formal analysis, methodology, validation, visualization, writing the original draft, writing – review, and editing. **Hideyuki Nakanishi**: data curation, investigation, methodology, resources. **Lisa Fujimura**: investigation, methodology. **Chie Kawai**: investigation, methodology. **Akima Harada**: data curation, investigation, methodology. **Kosuke Torigata**: formal analysis, methodology, validation, visualization. **Kenji Miki**: conceptualization, methodology, project administration, supervision. **Keiji Itaka**: conceptualization, methodology, project administration, resources, supervision, validation, writing – review, and editing. **Shigeru Miyagawa**: conceptualization, supervision.

## Funding

This study was supported by the Japan Society for the Promotion of Science (23K08253, 24KJ1659), Japan Agency for Medical Research and Development (25ak0101173s0205, JP223fa627002), Program on Open Innovation Platform with Enterprises, Research Institute and Academia (JPMJPF2202). This research was supported in part by Axcelead Inc.

## Conflicts of Interest

The authors declare no conflicts of interest.

## Supporting information

Supplementary Material

## Data Availability

The datasets presented in this study can be found in online repositories. The names of the repository/repositories and accession number(s) can be found below: https://www.ncbi.nlm.nih.gov/geo/info/linking.html, GSE304168.
