# Supplementary material for: Nanomicelle‐Based Multi‐mRNA Delivery Promotes Cardiac Repair After Myocardial Infarction
Source: Small Sci. 2026 May 23;6(5):e202500521. doi: 10.1002/smsc.202500521 (PMC13248758; doi:10.1002/smsc.202500521)
Supplement: Supplementary file 1 — Supplementary Material [file SMSC-6-e202500521-s001.pdf]

# Supplementary Files

## Nanomicelle-Based Multi-mRNA Delivery Promotes Cardiac Repair After Myocardial Infarction

| | | | Size (d.n... | % Intensity: | St Dev (d.n... |
|---|---|---|---|---|---|
| **Z-Average (d.nm):** | 89.01 | **Peak 1:** | 93.22 | 100.0 | 20.14 |
| **PdI:** | 0.079 | **Peak 2:** | 0.000 | 0.0 | 0.000 |
| **Intercept:** | 0.971 | **Peak 3:** | 0.000 | 0.0 | 0.000 |

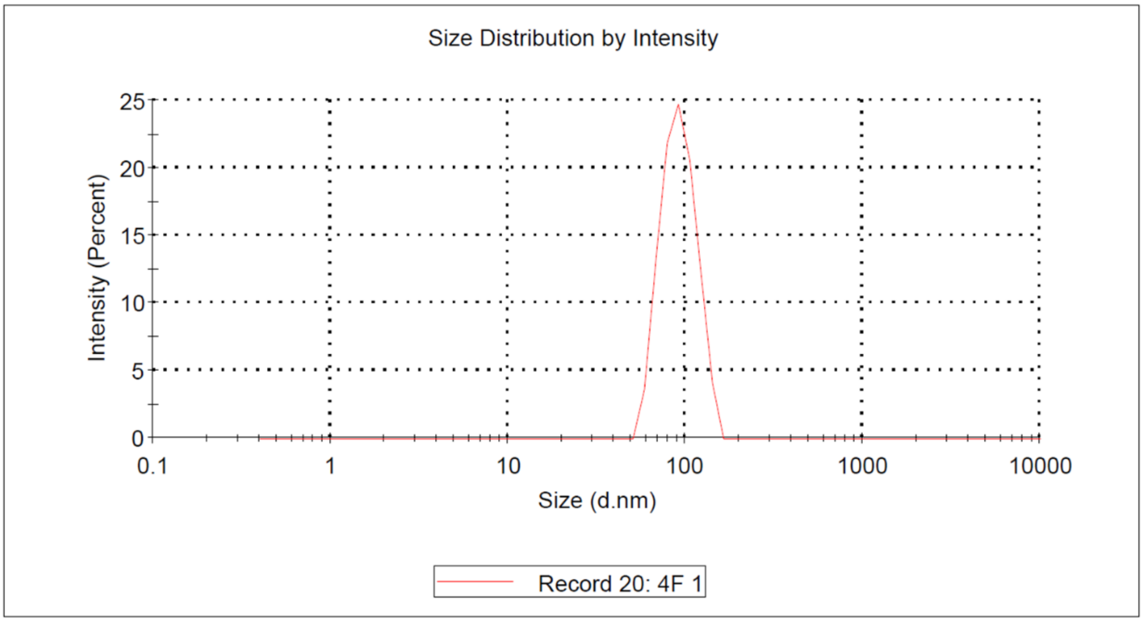


| | | | Mean (mV) | Area (%) | St Dev (mV) |
|---|---|---|---|---|---|
| **Zeta Potential (mV):** | 0.158 | **Peak 1:** | 0.158 | 100.0 | 5.77 |
| **Zeta Deviation (mV):** | 5.77 | **Peak 2:** | 0.00 | 0.0 | 0.00 |
| **Conductivity (mS/cm):** | 0.289 | **Peak 3:** | 0.00 | 0.0 | 0.00 |

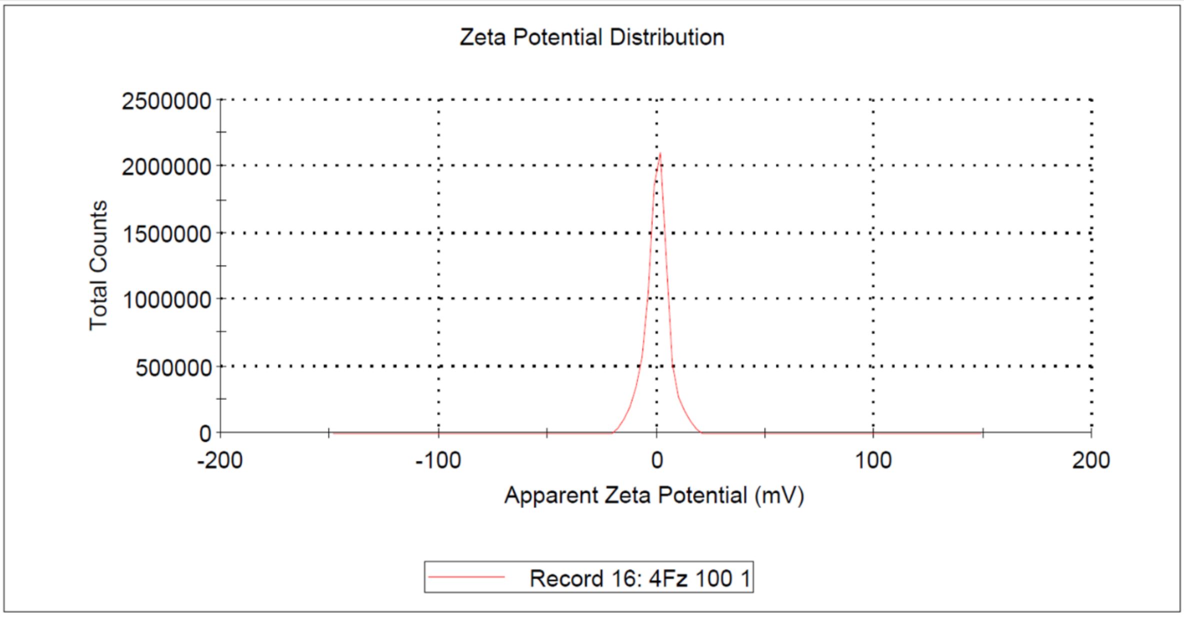


**Figure S1. Particle diameter and surface charge of polyplex nanomicelle measured by dynamic light scattering.**

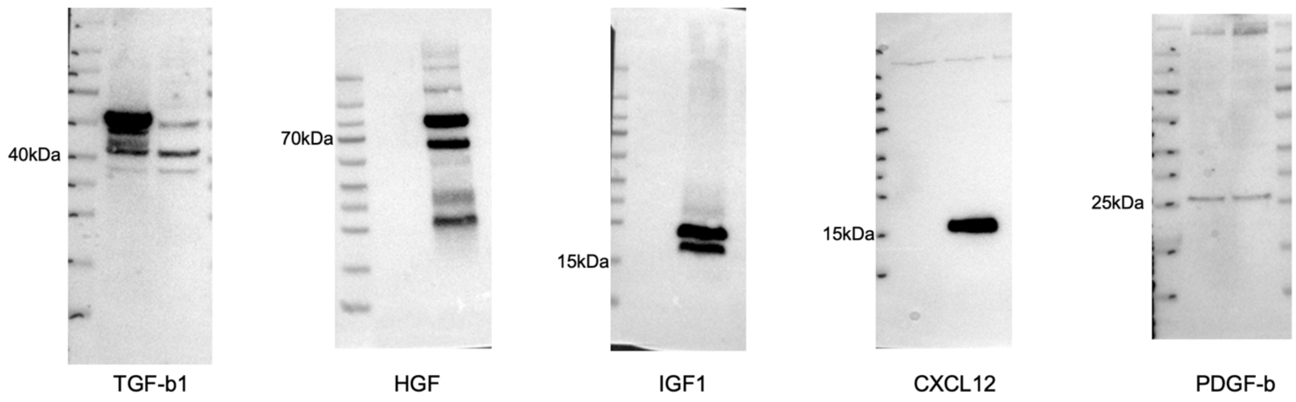


**Figure S2. Western blotting for the 5 factors mRNA.**

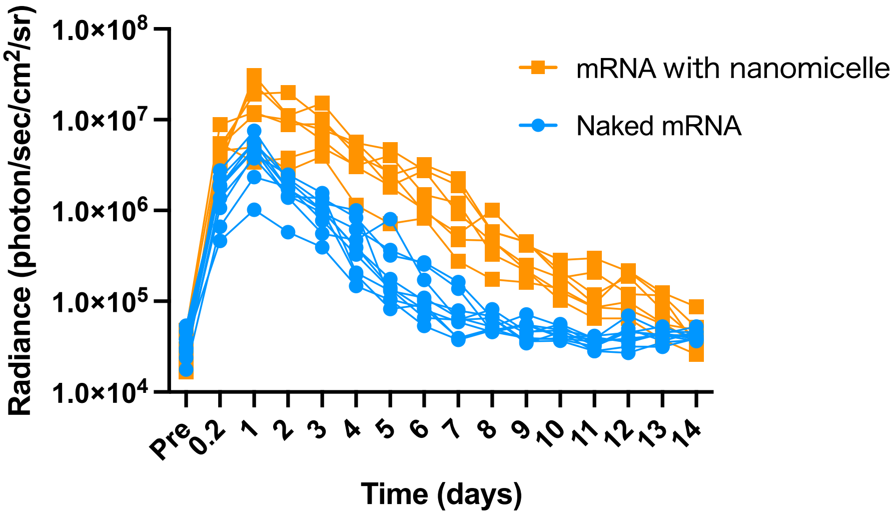


**Figure S3. Individual radiance profiles of mouse hearts in each group measured by IVIS through day 14**

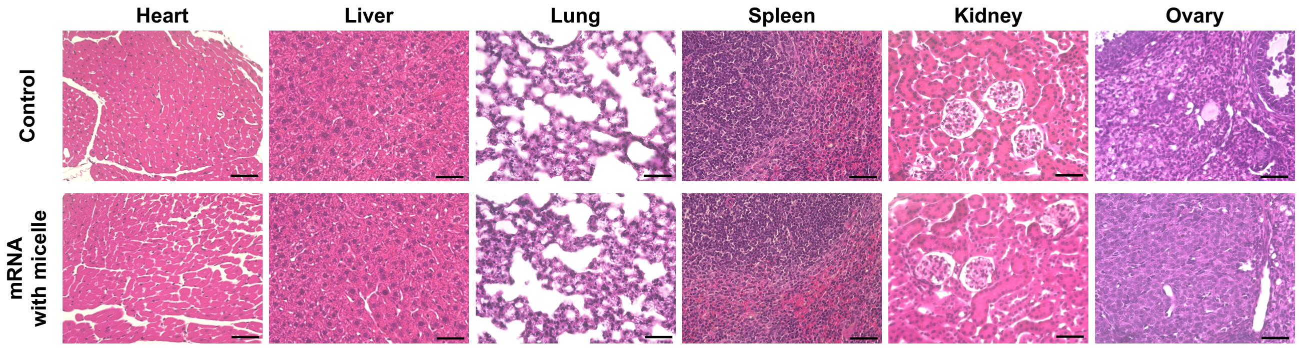


**Figure S4. Histological assessment of major organs.**
Hematoxylin and eosin (H&E) staining of organs harvested 24 h after intramyocardial administration of mRNA-loaded nanomicelles. No inflammatory cell infiltration or histological evidence of tissue injury was observed.

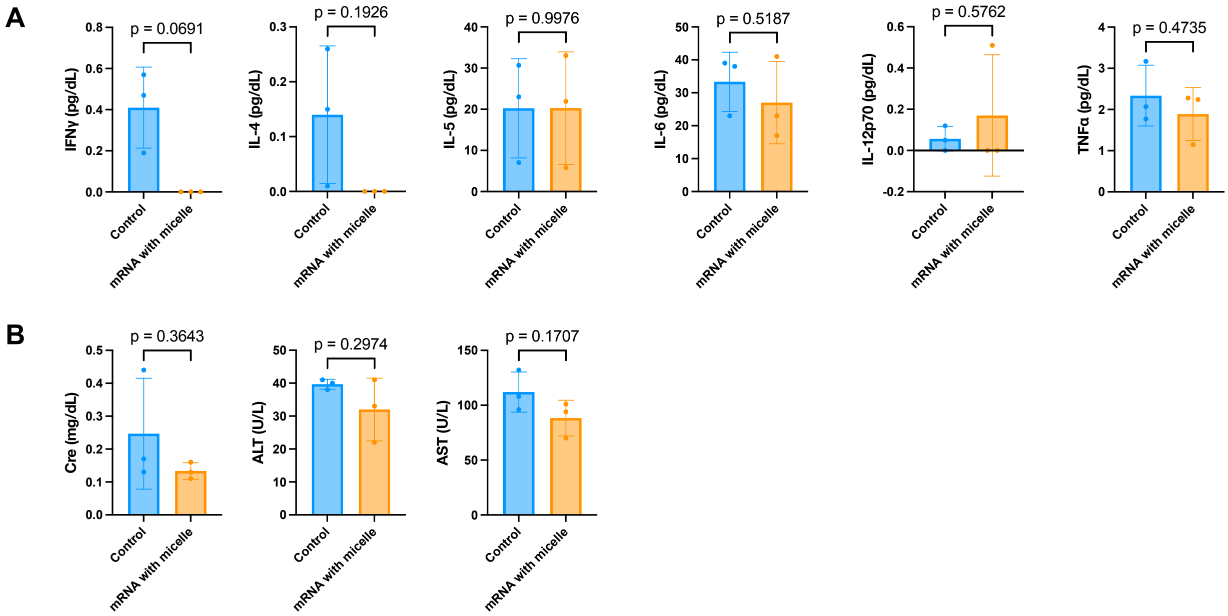


**Figure S5. Evaluation of Immunological Responses and Hepatic and Renal Function**
Inflammatory cytokines (**A**) and biochemical markers of hepatic and renal function (**B**) measured in blood samples collected 24 h after intramyocardial administration of mRNA-loaded nanomicelles.

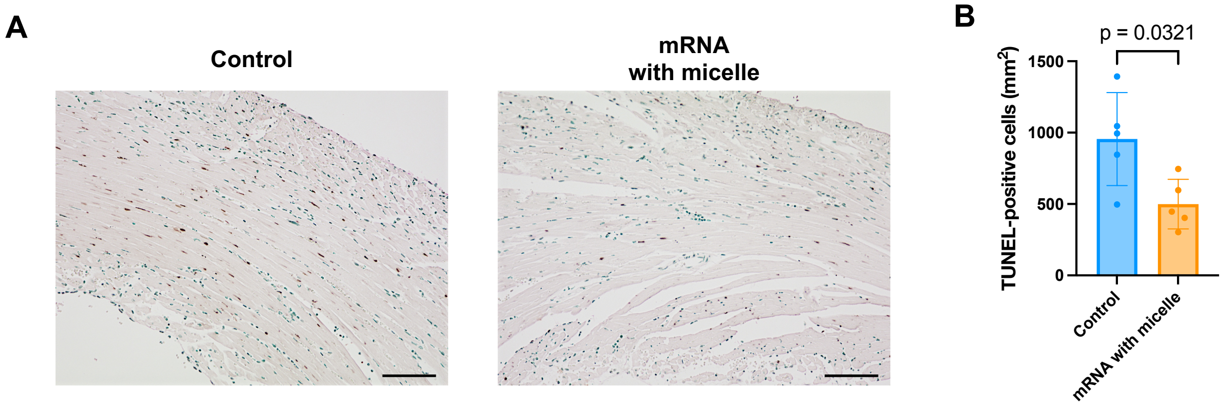


**Figure S6. TUNEL staining to assess cardiomyocyte death**
**A.** Representative TUNEL staining images for each group. Scale bars, 100 µm. **B.** Quantitative comparison of TUNEL-positive cell counts.

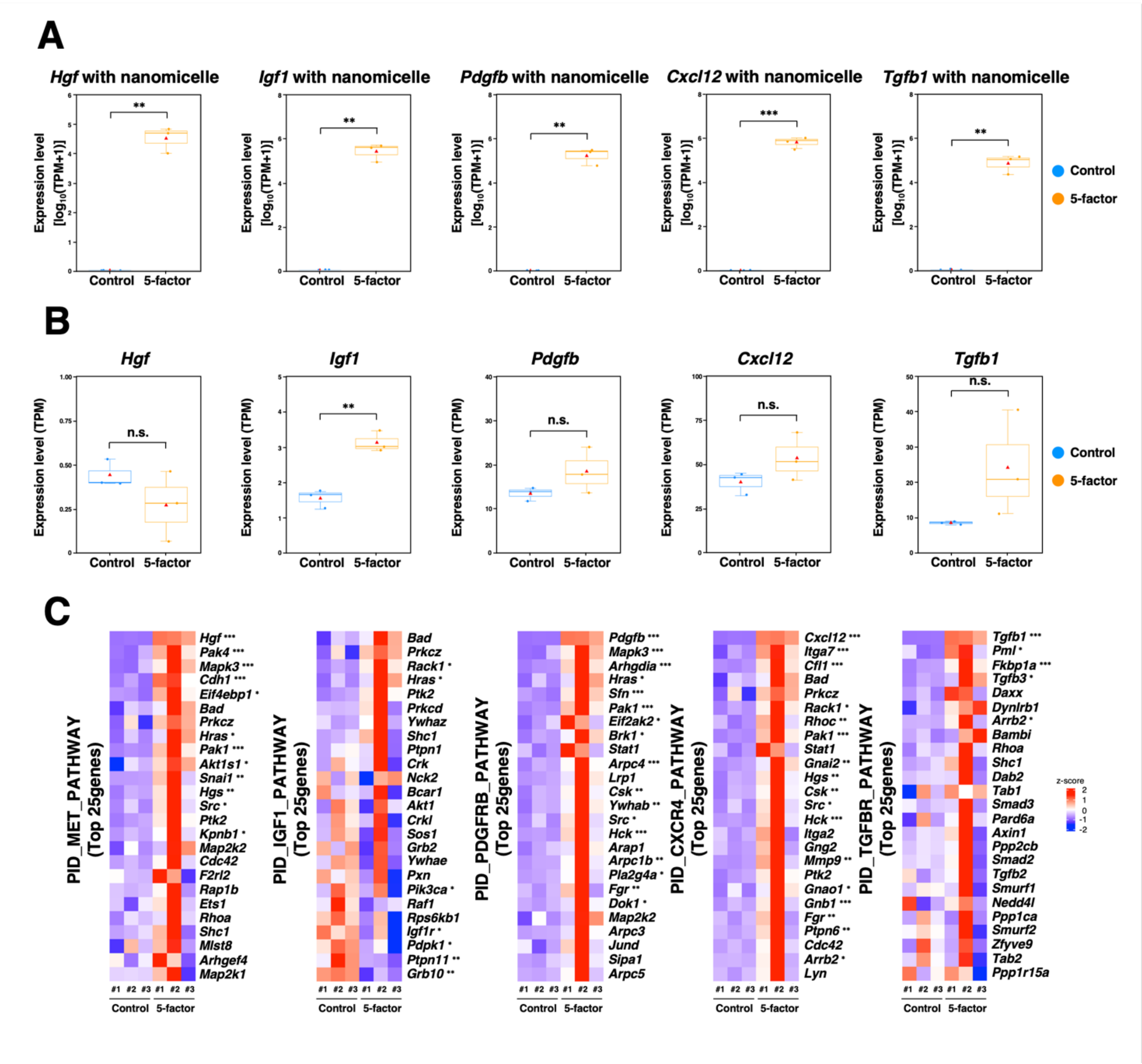


**Figure S7. 5-factor gene expression in RNA-Seq and the distribution of expression changes across each pathway.**

(**A**) Gene expression results of RNA-seq of exogenous 5-factor administered in nanomicelles are shown. (**B**) Displays the results of gene expression of endogenous 5-factor. In both (**A**) and (**B**), ***p* < 0.01 and ****p* < 0.001 indicate significant differences in Student's t-test when comparing the 5-factor to the control group. (**C**) The top 25 genes in the GSEA PID pathway associated with the 5-factor are shown as a heatmap. The heatmap shows the z-score of $log_2$FC (TPM + 1) in RNA-Seq. **q* < 0.05, ***q* < 0.01, ****q* < 0.001 indicate significant differences in DESeq2 when comparing the 5-factor to the control.

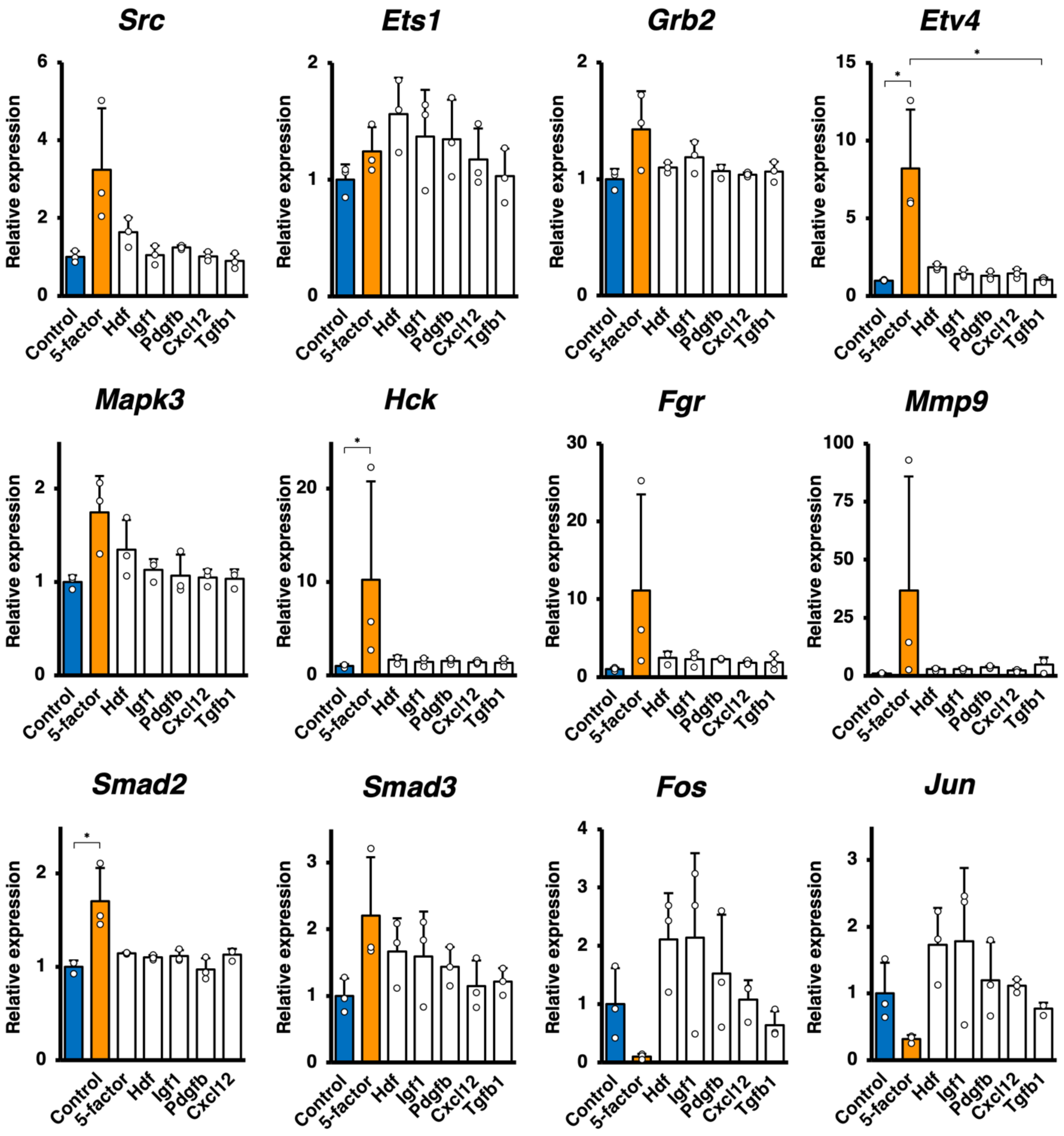


**Figure S8. Verification of gene expression changes in downstream genes of the 5-factor using RT-qPCR.**

Relative gene expression results for downstream factors of the 5-factor pathway, as determined by RT-qPCR, are shown. All data from the myocardial infarction model (Control), 5-factor treatment, and individual factor treatment were normalized by Gapdh expression and presented as relative values to the Control group. Data are shown as mean ± SD. Statistical analysis was performed using the Dunn-Bonferroni test for multiple comparisons. *$p < 0.05$ indicates a significant difference.

**Supplementary Table. Primers used for RT-qPCR.**

| Target gene | Primer types | Primer sequence | Length (bp) |
|---|---|---|---|
| *Gapdh* | Fw | AAGGTCATCCCAGAGCTGAA | 138 |
| | Rv | CTGCTTCACCACCTTCTTGA | |
| *Src* | Fw | GCTCTTCGGAGGCTTCAACT | 180 |
| | Rv | CACCAGTCTCCCTCTGTGTTA | |
| *Ets1* | Fw | CAAGCCGACTCTCACCATCA | 211 |
| | Rv | GACGTGGGTTTCTGTCCACT | |
| *Grb2* | Fw | AACATCCGTGTCCAGGAACC | 224 |
| | Rv | GGGTGACATAATTGCGGGGA | |
| *Etv4* | Fw | CGAGTGCCCTACACCTTCTG | 182 |
| | Rv | CTGTCCGGTACCTGAGCTTC | |
| *Mapk3* | Fw | GTCTCTGCCCTCGAAAACCA | 203 |
| | Rv | TGTCGAAGGTGAATGGCTCC | |
| *Hck* | Fw | AAGCTACACGCTGTGGTCTC | 171 |
| | Rv | CCTCTGCTCAATGAAGGCCA | |
| *Fgr* | Fw | CCCCCAACAAGGAACCAAGT | 187 |
| | Rv | GTAGCCATGCTCCACCTGTT | |
| *Mmp9* | Fw | CAGCCGACTTTTGTGGTCTTC | 208 |
| | Rv | CCTTTAGTGTCTGGCTGTCCA | |
| *Smad2* | Fw | ACGGTAGATCAGTGGGACAC | 175 |
| | Rv | CGCAGTTTTCGATTGCCTTGA | |
| *Smad3* | Fw | CTCAAGAAGACGGGGCAGTT | 151 |
| | Rv | ACAGGCGGCAGTAGATAACG | |
| *Fos* | Fw | TTTCAACGCCGACTACGAGG | 183 |
| | Rv | GCTGTCACCGTGGGGATAAA | |
| *Jun* | Fw | CGCCTGATCATCCAGTCCAG | 153 |
| | Rv | GGGAAGCGTGTTCTGGCTAT | |

**Supplementary Text. RNA sequences.**

Primer sequences

- Forward primer:
CAGTGAATTGTAATACGACTCACTATAAGGCGAATTAAGAGAGAAAAGAAGAG

- Reverse primer:
TTTTTTTTTTTTTTTTTTTTTTTTTTTTTTTTTTTTTTTTTTTTTTTTTTTTTTTTTTTTTTTT
TTTTTTTTTTTTTTTTTTTTTTTTTTTTTTTTTTTTTTTTTTTTTTTTTTTTTTCCTACTCAGGC
TTTATTCA

IVT template sequences (bold: T7 promoter, underllned: coding regions)

- mTGFb1
CAGTGAATTGTAATACGACTCACTATAAGGCGAATTAAGAGAGAAAAGAAGAGTAAGA
AGAAATATAAGACACCGGTCGCCACCATGCCTCCAAGCGGACTGAGACTGCTTCCTCTG
CTGCTGCCTCTGCCTTGGCTGCTGGTTCTTACACCTGGCAGACCTGCTGCCGGCCTGAGC
ACATGCAAGACCATCGACATGGAACTGGTCAAGAGGAAGAGAATCGAGGCCATCAGAG
GCCAGATCCTGAGCAAGCTGAGACTGGCTAGCCCTCCTAGTCAGGGCGAAGTTCCTCCT
GGACCTCTGCCTGAAGCTGTGCTGGCCCTGTACAACAGCACCAGAGATAGAGTGGCCGG
CGAGTCTGCTGATCCAGAGCCTGAACCTGAGGCCGACTACTACGCCAAAGAAGTGACC
AGAGTCCTGATGGTGGACCGGAACAACGCCATCTACGAAAAGACCAAGGACATCAGCC
ACAGCATCTACATGTTCTTCAACACCAGCGACATCCGCGAGGCCGTGCCTGAACCACCT
CTGCTGTCTAGAGCAGAGCTGAGGCTGCAGAGACTGAAGTCCAGCGTGGAACAGCACG
TGGAACTGTACCAGAAGTACAGCAACAACAGCTGGCGCTACCTGGGCAACAGACTGCT
GACCCCTACAGACACACCTGAGTGGCTGAGCTTCGACGTGACAGGCGTTGTCAGACAGT
GGCTGAATCAAGGCGACGGCATCCAGGGCTTCAGATTCAGCGCCCACTGTAGCTGCGAC
AGCAAGGACAACAAGCTGCACGTCGAGATCAACGGCATCAGCCCCAAGAGAAGAGGCG
ACCTGGGCACCATCCACGATATGAACAGACCCTTCCTGCTGCTGATGGCCACACCTCTG
GAAAGAGCCCAGCATCTGCACAGCTCCAGACATCGGAGAGCCCTGGACACCAACTACT
GCTTCAGCAGCACCGAGAAGAACTGCTGCGTGCGGCAGCTGTACATCGACTTCAGAAA
GGACCTCGGCTGGAAGTGGATCCATGAGCCTAAGGGCTACCACGCCAACTTTTGTCTGG
GCCCCTGTCCTTACATCTGGTCCCTGGATACCCAGTACTCCAAGGTCCTGGCTCTGTATA
ACCAGCACAACCCAGGCGCCAGCGCCTCTCCTTGTTGTGTGCCTCAAGCTCTGGAACCC
CTGCCTATCGTGTACTACGTGGGCAGAAAGCCCAAGGTCGAGCAGCTGAGCAACATGAT
CGTGCGGAGCTGCAAGTGCAGCTGAATCTAGACCTTCTGCGGGGCTTGCCTTCTGGCCA
TGCCCTTCTTCTCTCCCTTGCACCTGTACCTCTTGGTCTTTGAATAAAGCCTGAGTAGGA
AAAAAAAAAAAAAAAAAAAAAAAAAAAAAAAAAAAAAAAAAAAAAAAAAAAAAAAAAAA
AAAAAAAAAAAAAAAAAAAAAAAAAAAAAAAAAAAAAAAAAAAAAAAAAAAAAAAAAAA
AAAAAAA

- mHGF
CAGTGAATTGTAATACGACTCACTATAAGGCGAATTAAGAGAGAAAAGAAGAGTAAGA
AGAAATATAAGACACCGGTCGCCACCATGATGTGGGGAACAAAGCTGCTGCCCGTGCT
GCTGCTGCAACACGTTCTGCTTCATCTGCTGTTGCTGCACGTGGCCATTCCTTACGCTGA
GGGCCAGAAGAAGAGAAGAAACACCCTGCACGAGTTCAAGAAGTCCGCCAAGACCACA
CTGACCAAAGAGGACCCTCTGCTGAAGATCAAGACCAAGAAAGTGAACAGCGCCGACG
AGTGCGCCAACAGATGCATCAGAAACAGAGGCTTCACCTTCACATGCAAGGCCTTCGTG

TTCGACAAGAGCAGAAAGCGGTGCTACTGGTATCCCTTCAACAGCATGAGCAGCGGCGT
GAAGAAAGGCTTCGGCCACGAGTTCGACCTGTACGAGAACAAGGACTACATCCGGAAC
TGCATCATCGGCAAAGGCGGCAGCTACAAGGGCACCGTGTCTATCACCAAGAGCGGCA
TCAAGTGCCAGCCTTGGAACTCTATGATCCCTCACGAGCATAGCTTTCTGCCCAGCAGCT
ACAGAGGCAAGGACCTGCAAGAGAACTACTGCAGAAACCCCAGAGGCGAGGAAGGCG
GCCCTTGGTGTTTCACAAGCAACCCCGAAGTCCGCTACGAAGTGTGCGACATCCCTCAG
TGCAGCGAGGTGGAATGCATGACCTGTAACGGCGAGAGCTACAGGGGCCCTATGGACC
ACACAGAGAGCGGCAAGACCTGTCAGAGATGGGACCAGCAGACCCCTCACAGACACAA
GTTTCTGCCCGAGAGATACCCCGACAAGGGCTTCGACGACAACTACTGTAGGAACCCTG
ACGGCAAGCCCAGACCTTGGTGCTACACACTGGACCCTGACACACCCTGGGAGTACTGC
GCCATCAAGACATGTGCCCACAGCGCCGTGAACGAGACAGACGTGCCAATGGAAACCA
CCGAGTGCATCCAAGGCCAAGGCGAGGGCTATAGAGGCACCAGCAACACCATCTGGAA
CGGCATCCCTTGCCAAAGATGGGACTCTCAGTACCCTCACAAGCACGACATCACCCCTG
AGAACTTCAAGTGCAAGGATCTGAGGGAAAATTACTGCCGCAATCCTGACGGCGCCGA
GTCTCCATGGTGCTTCACAACAGACCCTAACATCAGAGTGGGCTACTGCTCTCAGATCC
CCAAGTGCGACGTGTCCTCTGGCCAGGACTGTTATCGCGGCAACGGCAAGAACTACATG
GGCAACCTGAGCAAGACCAGAAGCGGCCTGACCTGCAGCATGTGGGACAAGAACATGG
AAGATCTGCACCGGCACATCTTTTGGGAGCCCGACGCCAGCAAGCTGAACAAGAATTAC
TGTCGAAACCCCGACGACGACGCTCACGGACCCTGGTGTTATACAGGCAACCCTCTGAT
CCCTTGGGACTACTGCCCTATCAGCAGATGCGAGGGCGACACCACACCTACCATCGTGA
ACCTGGATCACCCCGTGATCAGCTGCGCCAAGACAAAGCAGCTGAGAGTGGTCAACGG
AATCCCCACACAGACCACCGTCGGCTGGATGGTGTCCCTGAAGTACAGAAACAAGCAC
ATCTGCGGCGGCAGCCTGATCAAAGAAAGCTGGGTGCTGACCGCCAGACAGTGCTTCCC
CGCTAGAAACAAAGACCTGAAGGACTACGAGGCCTGGCTGGGCATCCACGATGTTCAC
GAGAGAGGCGAAGAGAAGCGCAAGCAGATCCTGAACATCAGCCAGCTGGTGTACGGCC
CTGAGGGCTCTGATCTGGTGCTGCTGAAACTGGCCAGACCTGCCATCCTGGACAACTTC
GTGTCCACCATCGACCTGCCTAGCTACGGCTGCACAATCCCCGAAAAGACCACCTGTAG
CATCTACGGCTGGGGCTACACCGGACTGATCAACGCTGATGGCCTGCTGAGAGTCGCTC
ACCTGTACATCATGGGAAACGAGAAGTGCAGCCAGCACCACCAGGGCAAAGTGACCCT
GAACGAGAGCGAACTGTGTGCCGGCGCTGAGAAGATCGGATCTGGACCTTGTGAAGGC
GACTACGGCGGACCACTGATCTGCGAGCAGCACAAGATGAGAATGGTGCTGGGCGTGA
TCGTGCCTGGCAGAGGATGTGCTATCCCTAACAGACCCGGCATCTTCGTGCGCGTGGCC
TACTACGCTAAGTGGATTCACAAAGTCATCCTGACCTACAAGCTGTGAATCTAGACCTT
CTGCGGGGCTTGCCTTCTGGCCATGCCCTTCTTCTCTCCCTTGCACCTGTACCTCTTGGTC
TTTGAATAAAGCCTGAGTAGGAAAAAAAAAAAAAAAAAAAAAAAAAAAAAAAAAAAAAA
AAAAAAAAAAAAAAAAAAAAAAAAAAAAAAAAAAAAAAAAAAAAAAAAAAAAAAAAAAAA
AAAAAAAAAAAAAAAAAAAAAAAAAAAA

- mPDGFb

CAGTGAATTGTAATACGACTCACTATAAGGCGAATTAAGAGAGAAAAGAAGAGTAAGA
AGAAATATAAGACACCGGTCGCCACCATGAACAGATGCTGGGCTCTGTTCCTGCCTCTG
TGCTGCTACCTGAGACTGGTGTCTGCTGAGGGCGACCCCATTCCTGAGGAACTGTACGA
GATGCTGAGCGACCACAGCATCAGAAGCTTCGACGACCTGCAGAGACTGCTGCACAGA
GACAGCGTGGACGAGGATGGCGCTGAGCTGGACCTGAACATGACAAGAGCCCACAGCG
GCGTGGAACTGGAAAGCAGCTCTAGAGGCAGAAGAAGCCTGGGCTCTCTGGCTGCTGC
TGAGCCTGCTGTGATCGCCGAGTGCAAGACCAGAACCGAGGTGTTCCAGATCAGCAGA
AACCTGATCGACAGGACCAACGCCAACTTCCTCGTGTGGCCTCCTTGCGTGGAAGTGCA
GAGATGCAGCGGCTGCTGCAACAACAGAAACGTGCAGTGCAGAGCCAGCCAGGTGCAG
ATGAGGCCTGTGCAAGTGCGGAAGATCGAGATCGTGCGCAAGAAGCCCATCTTCAAGA
AAGCCACCGTGACACTGGAAGATCACCTGGCCTGCAAGTGTGAAACCGTGGTCACCCCT

AGACCTGTGACCAGATCTCCTGGCACCAGCAGAGAGCAGAGGGCCAAAACACCTCAGG
CCAGAGTGACCATCAGAACCGTGCGGATCAGAAGGCCTCCTAAGGGCAAGCACAGAAA
GTTCAAGCACACCCACGACAAGGCCGCTCTGAAAGAGACACTGGGCGCTTGAATCTAG
ACCTTCTGCGGGGCTTGCCTTCTGGCCATGCCCTTCTTCTCTCCCTTGCACCTGTACCTCT
TGGTCTTTGAATAAAGCCTGAGTAGGAAAAAAAAAAAAAAAAAAAAAAAAAAAAAAA
AAAAAAAAAAAAAAAAAAAAAAAAAAAAAAAAAAAAAAAAAAAAAAAAAAAAAAAA
AAAAAAAAAAAAAAAAAAAAAAAAAAAAAAAAA

- mIGF1

CAGTGAATTGTAATACGACTCACTATAAGGCGAATTAAGAGAGAAAAGAAGAGTAAGA
AGAAATATAAGACACCGGTCGCCACCATGGGAAAGATCAGCAGCCTGCCTACACAGCT
GTTCAAGATCTGCCTGTGCGACTTCCTGAAGATCAAGATCCACATCATGAGCAGCAGCC
ACCTGTTCTACCTGGCTCTGTGCCTGCTGACCTTCACCAGCTCTACAACCGCCGGACCTG
AGACACTGTGTGGCGCTGAACTGGTGGATGCCCTGCAGTTTGTGTGTGGCCCCAGAGGC
TTCTACTTCAACAAGCCTACAGGCTACGGCAGCAGCATCAGAAGGGCTCCTCAGACAGG
CATCGTGGACGAGTGCTGCTTCAGAAGCTGCGACCTGAGAAGGCTGGAAATGTACTGCG
CCCCTCTGAAGCCTACAAAGGCCGCCAGATCTATCAGAGCCCAGAGACACACCGACAT
GCCCAAGACACAGAAGTCCCCAAGCCTGAGCACCAACAAAAAGACCAAGCTGCAGAGA
AGGCGGAAGGGCAGCACCTTCGAGGAACACAAGTGAATCTAGACCTTCTGCGGGGCTT
GCCTTCTGGCCATGCCCTTCTTCTCTCCCTTGCACCTGTACCTCTTGGTCTTTGAATAAAG
CCTGAGTAGGAAAAAAAAAAAAAAAAAAAAAAAAAAAAAAAAAAAAAAAAAAAAAA
AAAAAAAAAAAAAAAAAAAAAAAAAAAAAAAAAAAAAAAAAAAAAAAAAAAAAAAA
AAAAAAAAAAAAAAAAAA

- mCXCL12

CAGTGAATTGTAATACGACTCACTATAAGGCGAATTAAGAGAGAAAAGAAGAGTAAGA
AGAAATATAAGACACCGGTCGCCACCATGGATGCTAAAGTGGTGGCCGTGCTGGCCCTG
GTTCTTGCCGCTCTGTGTATCTCTGACGGCAAGCCCGTGTCTCTGAGCTACAGATGCCCC
TGCAGATTCTTCGAGAGCCATATCGCCAGAGCCAACGTGAAGCACCTGAAGATCCTGAA
CACCCCTAACTGCGCTCTGCAGATCGTGGCCAGACTGAAGAACAACAACAGGCAAGTG
TGCATCGACCCCAAGCTGAAGTGGATCCAAGAGTACCTGGAAAAGGCCCTGAACAAAG
GCAGACGCGAGGAAAAAGTGGGCAAGAAAGAGAAGATCGGCAAGAAGAAGCGCCAGA
AGAAGAGAAAGGCCGCTCAGAAGAGGAAGAACTGAATCTAGACCTTCTGCGGGGCTTG
CCTTCTGGCCATGCCCTTCTTCTCTCCCTTGCACCTGTACCTCTTGGTCTTTGAATAAAGC
CTGAGTAGGAAAAAAAAAAAAAAAAAAAAAAAAAAAAAAAAAAAAAAAAAAAAAAA
AAAAAAAAAAAAAAAAAAAAAAAAAAAAAAAAAAAAAAAAAAAAAAAAAAAAAAAA
AAAAAAAAAAAAAAAAA
